# Transcriptomic Signatures of *Trichomonas vaginalis* Isolates That Exhibit Low, Intermediate, and High In Vitro Resistance to Metronidazole

**DOI:** 10.3390/microorganisms14061314

**Published:** 2026-06-12

**Authors:** Keonte J. Graves, Colin Reily, W. Evan Secor, Jan Novak, Christina A. Muzny

**Affiliations:** 1 Division of Infectious Diseases, Department of Medicine, University of Alabama at Birmingham, Birmingham, AL 35233, USA; keontegraves@uabmc.edu; 2 Division of Nephrology, Department of Medicine, University of Alabama at Birmingham, Birmingham, AL 35233, USA; creily@uab.edu; 3 Department of Microbiology, University of Alabama at Birmingham, Birmingham, AL 35233, USA; jannovak@uab.edu; 4 Division of Parasitic Diseases and Malaria, Centers for Disease Control and Prevention, Atlanta, GA 30329, USA; was4@cdc.gov

**Keywords:** *Trichomonas vaginalis*, transcriptomics, RNA-sequencing, drug susceptibility, drug resistance

## Abstract

As part of efforts to identify genes associated with *Trichomonas vaginalis* resistance to 5-nitroimidazole drugs, thirty cryopreserved *T. vaginalis* isolates were revived and grown using Diamond’s TYM medium. Minimum lethal concentrations (MLCs) for metronidazole (MTZ), tinidazole (TDZ), and secnidazole (SEC) were determined using a drug susceptibility assay. Transcriptome profiling was performed for 15 MTZ-sensitive (MTZ-S, MLC < 50 µg/mL) and 15 MTZ-resistant (MTZ-R, MLC ≥ 50 µg/mL) isolates using next-generation RNA sequencing. Bioinformatics analyses identified differentially expressed genes (DEGs). Among the MTZ-R isolates, six exhibited low MLCs of 50 µg/mL, five had intermediate MLCs between 100 and 200 µg/mL, and four had high MLCs ≥ 400 µg/mL. Differential gene expression analysis identified 28, 140, and 73 significantly altered genes in low-, intermediate-, and high-level MTZ resistance groups, respectively, with predominantly upregulated expression patterns. The SEC-resistant (SEC-R) isolates exhibited 136 differentially expressed genes, whereas the TDZ-resistant (TDZ-R) isolates showed minimal transcriptional changes. Focused analyses of iron transport pathways revealed reduced expression of ZIP-family iron import genes, particularly *TvZIP4* (TVAG_273550), the strongest predictor of resistance in elastic-net modeling (AUC = 0.795). Resistant isolates also demonstrated coordinated upregulation of iron–sulfur cluster assembly and hydrogenosomal protein-import pathways. Weighted gene co-expression network analysis (WGCNA) identified multiple resistance-associated transcriptional modules correlated with MTZ and SEC MLCs. A comparative transcriptomic–proteomic analysis revealed concordant upregulation of iron–sulfur cluster machinery but discordant regulation of hydrogenosomal cargo proteins, likely supporting a post-transcriptional restriction model. These findings provide a broader mechanistic framework for understanding 5-nitroimidazole resistance in *T. vaginalis* and identifying candidate biomarkers and pathways that may support future therapeutic and diagnostic development.

## 1. Introduction

*Trichomonas vaginalis*, a microaerophilic parasitic protozoan, is the causative agent of trichomoniasis. *T. vaginalis* is the most common curable non-viral sexually transmitted infection (STI) worldwide and simultaneously one of the more underappreciated STIs [1,2]. The majority of *T. vaginalis* infections are asymptomatic, with the infection being more commonly clinically apparent in women than in men. Symptomatic infections can present in various ways: vaginitis, urethritis, malodorous and discolored vaginal discharge, urethral discharge, severe itching, painful sexual intercourse, and painful urination [3]. *T. vaginalis* infections disproportionately affect African Americans [4] and have also been associated with multiple adverse health outcomes: increased risk of acquisition of HIV and other STIs, infertility, pre-labor rupture of membranes, preterm birth, and low birth weight, and increased risk of cervical cancer related to co-infection with human papilloma virus [5].

**Figure 1:**
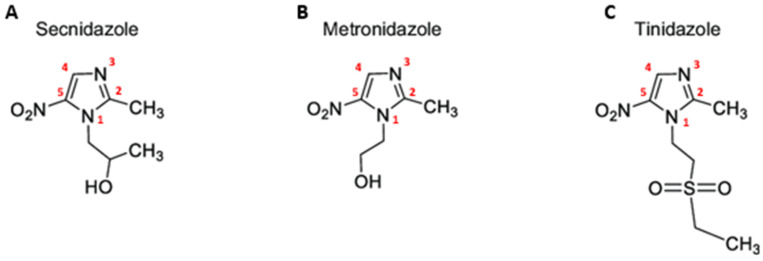
Molecular structures of the three FDA-approved 5-nitroimidazoles used to treat *T. vaginalis* infections. All three 5-nitroimidazoles share a common imidazole ring structure with a methyl group at position (2) and a nitro group at position (5). They vary in the groups attached to the nitrogen at position (1): (**A**) Secnidazole [SEC], hydroxypropyl group; (**B**) Metronidazole [MTZ], 2-hydroxyethyl group; (**C**) Tinidazole [TDZ], ethylsulfonylethyl side chain. Atom numbering scheme of 5-nitroimidazole ring (red numerals).

The treatment options for *T. vaginalis* infections in the United States come from a sole class of FDA-approved drugs, the 5-nitroimidazoles: metronidazole (MTZ), tinidazole (TDZ), and secnidazole (SEC) (Figure 1). MTZ has been the most common drug treatment for *T. vaginalis* infections since its development in the early 1960s [6]. However, resistance to MTZ was observed only a few years after its introduction [7]. Such a rapid appearance of treatment failure may suggest that resistance is encoded in the genome of some *T. vaginalis* isolates, rather than arising de novo; however, additional data are needed. The true prevalence of 5-nitroimidazole resistance in *T. vaginalis* is not known due to the lack of national surveillance programs, as it is not a reportable disease [8,9]. Thus, prevalence data on resistance are only available from small studies that suggest it occurs in approximately 4–10% of *T. vaginalis* infections [10,11]. This picture is further complicated as *T. vaginalis* resistance to 5-nitroimidazoles may be relative and not absolute. For example, *T. vaginalis* infections unresponsive to standard doses of MTZ may be cured by increasing the dosage and duration of treatment [12]. However, significant side effects in patients receiving high-dose therapy are a major limiting factor.

The 5-nitroimidazoles are prodrugs that must be reduced to be activated. They all share a common base imidazole ring structure with different modifications of nitrogen atoms at position 1 of the imidazole ring (Figure 1). The continued reliance on a single class of drugs for *T. vaginalis* treatment could possibly lead to an increase in the prevalence of resistant isolates to MTZ and further drive cross-resistance to the other 5-nitroimidazoles. Current methods of laboratory testing for drug resistance in trichomonas isolates consist of growing parasites in increasing drug concentrations to determine the minimal lethal concentration (MLC) leading to parasite death. The isolation, growth, and testing of parasites is time-consuming, and, to our knowledge, the Centers for Disease Control and Prevention (CDC) and the University of Alabama at Birmingham (UAB) STI Research Laboratory are the only locations in the United States where Clinical Laboratory Improvement Amendments (CLIA)-approved testing are presently performed.

A previous study conducted by our team investigated the transcriptomic profiles of a small number of *T. vaginalis* isolates (*n* = 8) [13]. The goal of that work was to gain a better understanding of *T. vaginalis* 5-nitroimidazole resistance mechanisms and attempt to identify genetic markers that could lead to future assays for more rapid MTZ-resistance detection and novel treatment options. The results from that study identified 304 differentially expressed genes (DEGs) from four MTZ-resistant and four MTZ-sensitive *T. vaginalis* isolates. Significant downregulation of genes encoding translational machinery and upregulation of genes encoding surface proteins (Leucine-rich repeat [LRR] proteins) involved in cytoadhesion, as well as dysregulated expression of “resistance-related” genes, were described for MTZ-resistant *T. vaginalis* isolates. In the present study, we have now expanded the number of isolates examined to determine whether MTZ-resistant (*n* = 15) vs. sensitive *T. vaginalis* (*n* = 15) clinical isolates differed in their expression of genes encoding virulence factors (i.e., cytotoxic proteins, proteases/peptidases, adhesion proteins, and antigenic surface proteins), stratified by MTZ susceptibility status (low, intermediate, and high). Furthermore, MLC values for MTZ, TDZ, and SEC were used for weighted gene co-expression network analyses (WGCNAs) to group genes into clusters (called “modules”) based on similar expression patterns. Additionally, we cross-compared our transcriptomic data against published proteomic data [14] and identified pathways associated with 5-nitroimidaloze resistance.

## 2. Materials and Methods

### 2.1. Selection of T. vaginalis Isolates and Culture Growth

**Figure 2:**
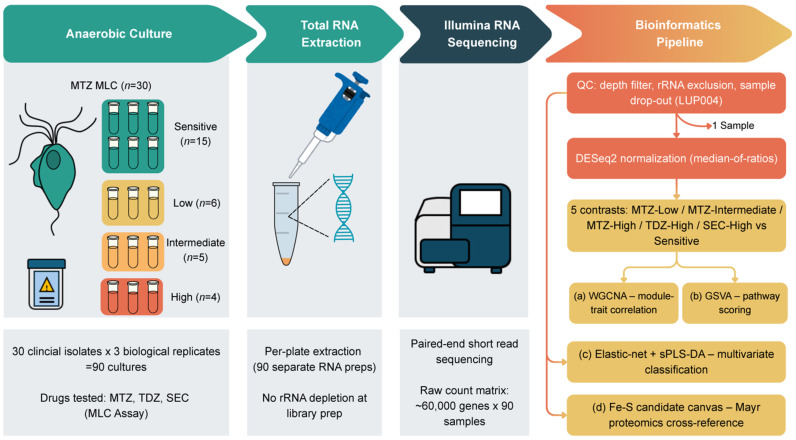
Schematic of *T. vaginalis* experimental and bioinformatic workflow. Anaerobic Culture: MTZ resistance status (low, intermediate, and high). Bioinformatics Pipeline: Isolate *LUP004* was excluded from analysis due to low read count.

Thirty cryopreserved *T. vaginalis* isolates obtained from biorepositories at UAB and CDC were used in this study. The isolates were selected to represent the varying resistance profiles encountered in both laboratory and clinical settings. Half of the isolates were MTZ-sensitive (*n* = 15, MLC ≤ 50 µg/mL), and the other half were MTZ-resistant (*n* = 15). The MTZ-resistant isolates were further divided into high- (*n* = 4, MTZ MLC ≥ 400 µg/mL), intermediate- (*n* = 5, MTZ MLC between 100 and 200 µg/mL), and low-level (*n* = 6, MTZ MLC = 50 µg/mL) MLC phenotypes (Figure 2). Isolates were obtained from the CDC under determination #CGH-LSDB-3/6/23-def6d. UAB isolates were obtained from previous studies during which written informed consent was obtained to use stored isolates for future research; UAB Institutional Review Board Protocol (IRB) Numbers: #300007385, #130425010, and #300008770, respectively.

The isolates were revived and grown in triplicate using an in-house Diamond’s Trypticase–Yeast–Maltose (TYM) medium. The cultures were initially placed into Mitsubishi anaerobic chambers with an AnaeroPack-Anaero pouch (Thermo Fisher Scientific, Waltham, MA, USA) and incubated between 33 and 37 °C for 3–5 days. Cultures were subsequently passed into fresh Diamond’s TYM media with added 100X Penicillin–Streptomycin–Amphotericin B antibiotic cocktail (MP Biomedicals, Solon, OH, USA) every other day to prevent yeast or bacterial contamination.

### 2.2. T. vaginalis 5-Nitroimidazole Drug Susceptibility Testing Assay

The MLCs for each *T. vaginalis* isolate were determined using a modified drug susceptibility assay for MTZ, TDZ, and SEC [15]. The isolates were grown in triplicate using our in-house Diamond’s TYM media in 96-well plates (Thermo Fisher Scientific, Waltham, MA, USA). The 5-nitroimidazoles were serially diluted across the plates from a high (400 µg/mL) to low (0.2 µg/mL) final drug concentration. The plates were then incubated under aerobic conditions between 33 and 37 °C for 48–56 h and observed using an inverted microscope to determine their respective MLCs based on trichomonad motility.

### 2.3. Extraction of Total RNA and Next-Generation Sequencing

*T. vaginalis* cultures were grown in triplicate from a single source and centrifuged at 2200 rpm for 10 min to form cell pellets. The supernatant was then removed and replaced with 1 mL of sterile phosphate-buffered saline (PBS). Total RNA was extracted from the PBS-washed *T. vaginalis* pellets using the RNAqueous™ Total RNA Isolation Kit (Invitrogen, Carlsbad, CA, USA). *T. vaginalis* cell pellets were transferred into 2 mL microcentrifuge tubes along with 1 mL of Trizol. The tubes were incubated at room temperature for 5 min. Next, 200 μL of chloroform was added to the tubes. The tubes were vigorously shaken for 15 s and allowed to incubate at room temperature for 10 min. Samples were then centrifuged (12,000× *g*) for 15 min. The aqueous layer was transferred to a new 2 mL microcentrifuge tube to which 500 μL of isopropanol was added before the tubes were incubated for 10 min at room temperature followed by centrifugation (12,000× *g*) for 8 min. The supernatant was carefully removed, and the RNA pellet washed using 1 mL of 75% ethanol and centrifuged (12,000× *g*) for 5 min. The ethanol was removed, and the RNA pellet was allowed to dry for 2–3 min before 30 μL of distilled water was added to dissolve the RNA pellet. The RNA pellets were incubated at 55 °C in a heating block for 15 min to further enhance RNA solubilization (Figure 2). The samples were subsequently stored at −80 °C until they were ready to be sent to Azenta Life Sciences (Burlington, MA, USA) for next-generation RNA sequencing (RNA-Seq). Counts were averaged prior to differential gene expression and grouped cohort analyses.

RNA isolation and purification processes did not use ribosomal RNA (rRNA) depletion. Therefore, we found that rRNAs accounted for approximately 49% of mapped reads, with a single 16s rRNA (TVAG 540780) responsible for approximately 35% of all reads. This issue was addressed at the normalization step for DEG analysis and by removal for WGCNA input matrix.

Next-generation RNA sequencing raw and curated data for this study are available at the NCBI Gene Expression Omnibus (GEO) under accession number GSE326939.

### 2.4. Bioinformatics and Statistical Analyses

Read counts were averaged across each isolate’s biological replicates to create a single dataset per isolate. Isolate *LUP004* was excluded prior to analysis due to low read count (50,000 vs. ~5 × 10^6^ average), leaving 29 total independent biological data sets (Figure 2). Differential expression analysis was performed using DESeq2 (v1.40) for all samples. Median of ratios was used for normalization to handle the rRNA confounding variable, followed by the Wald test with Benjamini–Hochberg false discovery rate (FDR) correction. Genes were considered significant at padj < 0.05 and log2 fold change ≥ 1. MTZ-Low/Intermediate/High, SEC-High (MLC ≥ 50), and TDZ-High (MLC ≥ 50) were compared to MTZ-sensitive. See Figure 2 for analytical pipeline.

### 2.5. Weighted Gene Co-Expression Network Analysis (WGCNA) Methods

WGCNA was performed using variance stabilizing transformed (VST) expression matrix from the DESeq2 dataset. Structural rRNA genes were excluded from the input, and the top 10,000 most variable genes were conserved for network construction. Signed network was used for module constructions with a soft thresholding power selected to meet scale-free topology of R2 ≥ 0.85, with a minimum module of 30 and cut height of 0.25. Modules were considered significant at pad < 0.05 for at least one trait. To assess module stability and overfitting, we ran leave-one-out (LOO; one isolate drop) and 100 bootstrap rebuilds. All sampling runs used identical parameters to the reference network. For each resampled network, we computed (A) the WGCA module preservation summary statistic, with the original network as reference and each resampled network as test set (100 permutations), (B) the recovery rate of each reference hub gene (kME > 0.80) as a hub gene of the resampled network, and (C) the best-Jaccard overlap of every reference module against the resamples modules. Z-summary > 10 indicates strong module preservation (Figure S1).

### 2.6. Curated Iron/Redox Pathway Gene Set Analysis

To analyze iron-redox gene pathways, a curated set was constructed from the TrichDB annotations database [16]. Three definitions were used. An annotation-only iron/redox set of 426 genes was made by systematic keyword search of the TrichDB dataset using descriptions across iron, redox, hydrogenosomal, ferredoxin, thioredoxin, peroxidase, oxidoreductase, NAD(P)H, flavodoxin, and metal transport terms. This list was used to score each isolate using Gene Set Variation Analysis (GSVA, gseapy v1.1, ssgsea method; Figure S2). Second, a proteomics subset (n = 88) of significant proteins were obtained from the Mayr et al. 2024 [14] published dataset that met significance across 2 mass spectrometry runs, and cross-analyzed for concordance across our GSVA results (Figure S3). Third, a dedicated 29-gene iron transport subset gene list was generated to test the iron handling pathway changes (Figure S4).

Multivariate analysis was performed using the 426 iron/redox list using sparse partial least squares discriminant analysis (sPLS-DA) and elastic-net logistic regression (scikit-learn v1.3). Training was performed using leave-one-out cross-validation for each of the 29 isolates, with 100 bootstrap stability selection resampling. This was performed on a continuous log2 for MTZ MLC and the top 5000 variance gene set vs. group level resistance.

Differential gene correlation analysis (DGCA) was performed on the iron/redox gene list to identify pairwise gene–gene correlations. We used a Z-score differential correlation test with Benjamini–Hochberg FDR correction (Figure S5A) and the permutation test to evaluate module-level correlation rewiring for the trait-significance WGCNA modules (Figure S5B).

### 2.7. Cross-Comparison with Published Proteomic Data

Gene mapping between the proteomic (*n* = 88) and transcriptomic datasets of significant DEGs and the TrichDB was performed to validate the correct correspondence. Proteomic fold changes were converted to signed log2 scale, preserving the sign for downregulated proteins where the input FC was negative. Concordance was defined as a matching direction of fold change across both the proteomic and transcriptomic platforms. The iron transport subset was further analyzed in a direction-stratified manner, separately for Mayr-UP versus Mayr-DOWN proteins [14], to test whether transcript–protein concordance differed systematically by direction. A Mann–Whitney U test was used to compare per-gene mean transcript log2 fold change between an additional iron–sulfur cluster assembly machinery group subset and a hydrogenosomal cargo group. Functional categories were assigned based on TrichDB and UniProt product descriptions.

### 2.8. Statistical Reporting Conventions

Differential gene expression analysis used Benjamini–Hochberg with FDR-adjusted *p*-values (padj) < 0.05 was considered significant. Set-level claims (GSVA scores, subcategory-level Wilcoxon signed-rank tests, continuous trait correlations on a pre-specified gene set) and per-gene claims within a pre-specified candidate family use the uncorrected *p*-value of the relevant single test (Wilcoxon signed-rank, Mann–Whitney U, or Pearson correlation), since the genome-wide multiple-testing burden does not apply to a single pre-specified test.

## 3. Results

### 3.1. Distribution of 5-Nitroimidazole Minimum Lethal Concentrations

MTZ-S isolates had MTZ MLCs ranging from 0.4 µg/mL to 25 µg/mL (Table 1). Among the MTZ-R isolates, six (40%) exhibited low-level MLCs (50 µg/mL), five (33%) intermediate MLCs (100 µg/mL–200 µg/mL), and four (27%) high MLCs (≥400 µg/mL). Of the 15 MTZ-R isolates, 13 (87%) were also resistant to TDZ (MLC ≥ 6.3 µg/mL) and 11 (73%) were resistant to SEC (MLC ≥ 12.5 µg/mL) in vitro. Additionally, 10 (67%) of the MTZ-resistant isolates showed cross-resistance to all three 5-nitroimidazoles.

### 3.2. Identification of Differentially Expressed Genes by MTZ Resistance Status

**Figure 3:**
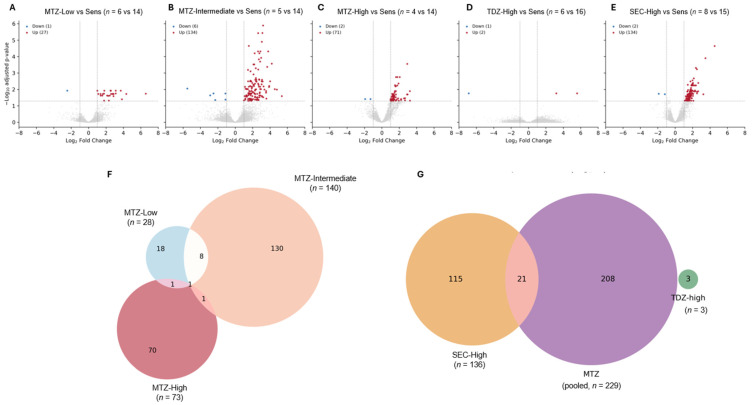
Differential gene expression across resistant MLC categories. (**A**–**E**) Five-panel volcano plot showing log2 fold change (*x*-axis) against log10(adjusted *p*-value) (*y*-axis) for MTZ-Low, MTZ-Intermediate, MTZ-High, TDZ-High, and SEC-High resistance contrasts versus *T. vaginalis* MTZ-sensitive controls. The significance threshold (padj < 0.05, log2FC ≥ 1) is shown as a dashed horizontal line. Significantly differentially expressed genes (DEGs) are color-coded by direction (red = upregulated in resistance, blue = downregulated, grey = not significant). DEG counts per contrast: MTZ-Low *n* = 28 (27 up/1 down); MTZ-Intermediate *n* = 140 (134 up/6 down); MTZ-High *n* = 73 (71 up/2 down); TDZ-High *n* = 3 (2 up/1 down); SEC-High *n* = 136 (134 up/2 down). (**F**) Three-way Venn diagram showing the overlap of significant DEGs among MTZ-Low, MTZ-Intermediate, and MTZ-High contrasts. Overlapping regions indicate genes that are significantly differentially expressed at multiple MLC levels. (**G**) Three-way Venn diagram showing the overlap of significant DEGs across the three 5-nitroimidazole-resistance contrasts (MTZ-pooled, TDZ-High, SEC-High). MLC, minimum lethal concentration; MTZ, metronidazole; TDZ, tinidazole; SEC, secnidazole; FC, fold change; padj, Benjamini–Hochberg adjusted *p*-value.

The transcriptomic profiling comparing the gene expression patterns between MTZ-R and MTZ-S *T. vaginalis* isolates identified several differentially expressed genes that could be associated with resistance. Furthermore, the MTZ-resistant *T. vaginalis* isolates exhibited altered expression of these genes in all isolates with MLCs ≥ 50 ug/mL. We have focused on the assessment of DEGs in the isolates with high vs. intermediate vs. low MLC values for MTZ (Figure 3).

#### 3.2.1. Differential Gene Expression in Isolates with Low-MTZ-Resistance MLCs

The MTZ-Low resistant group showed 28 significantly differentially expressed genes compared to MTZ-sensitive isolates (Figure 3A). Of the 28 DEGs, 27 were upregulated. Full gene-level results are in Table S1.

#### 3.2.2. Differential Gene Expression in Isolates with Intermediate MTZ MLCs

The MTZ-Intermediate resistant group showed 140 significantly differentially expressed genes compared to MTZ-sensitive isolates (Figure 3B). Of the 140 DEGs, 134 genes were upregulated. Full gene level results are in Table S2.

#### 3.2.3. Differential Gene Expression in Isolates with High MTZ MLCs

The MTZ-High resistant group showed 73 significantly differentially expressed genes compared to MTZ-sensitive isolates (Figure 3C). The direction of the DEGs was predominantly upward, at 71 out of 73. Full gene-level results are in Table S3.

#### 3.2.4. Cross-Drug Differential Gene Expression Overlap Among 5-Nitroimidazoles

TDZ-High vs. MTZ-sensitive showed 3 significant DEGs (Figure 3D, Table S4), and SEC-High vs. MTZ-sensitive showed 136 significant DEGs (134 upregulated; Figure 3E, Table S5). The TDZ contrast produced the smallest number of DEGs, partially due to high in-group heterogeneity. The cross-drug intersection analysis consisted predominantly of upregulated genes (Figure 3G).

### 3.3. Iron Transport Restriction in Resistant Isolates

**Figure 4:**
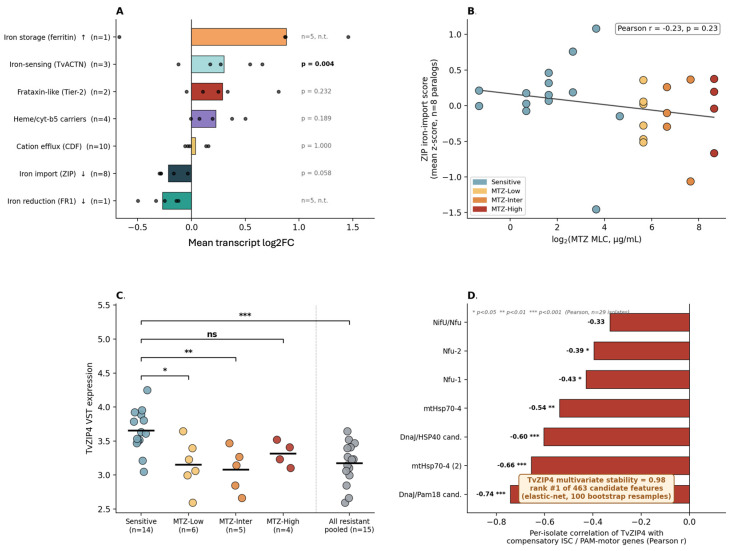
Iron handling and transport regulation correlates with resistance. (**A**) Mean transcript log2 fold change by mechanistic subcategory of the curated 29-gene iron transport set. Horizontal bars show the pooled mean log2FC across all member genes and all five resistance contrasts; small dots overlaid on each bar show per-contrast means. Bars are colored by functional direction (teal = import/access; mustard = export; orange = storage; purple = redox carriers; light blue = sensors; red = donor; up = upregulated; down = downregulated). The *p*-value to the right of each bar is from a Wilcoxon signed-rank test against zero on the pooled gene × contrast log2FC values. “n = k, n.t.” indicates subcategories with too few observations to test. (**B**) ZIP-family iron import score (mean z-score across the 8 ZIP/SLC39 paralogs) versus log2(MTZ MLC). Each point is one isolate, colored by resistance group (*n* = 14 sensitive, 6 MTZ-Low, 5 MTZ-Intermediate, 4 MTZ-High). The black trend line is from linear regression. The annotation shows the Pearson correlation against MTZ MLC. (**C**) Per-isolate variance stabilizing transformed (VST) *TvZIP4* (TVAG_273550/EAY07671) expression across the sensitive and three resistance groups and a pooled-resistant fifth column. Each point is one isolate; horizontal black bars indicate group means. Significance brackets show pairwise Mann–Whitney U tests against the Sensitive group at increasing y-positions: Sensitive vs. MTZ-Low *p* = 0.015 (*), sensitive vs. MTZ-Intermediate *p* = 0.003 (**), sensitive vs. MTZ-High *p* = 0.061 (ns), sensitive vs. all-resistant pooled *p* = 0.0008 (***). (**D**) Per-isolate Pearson correlation of *TvZIP4* expression with seven candidate compensatory iron–sulfur cluster (ISC) assembly and PAM motor genes (*n* = 29 isolates). Bars show correlation coefficients (Pearson’s r); asterisks denote significance (* *p* < 0.05, ** *p* < 0.01, *** *p* < 0.001). ZIP, Zrt-/Irt-like Protein; SLC39, solute carrier family 39; PAM, presequence-translocase-associated motor; ISC, iron–sulfur cluster.

**Figure 5:**
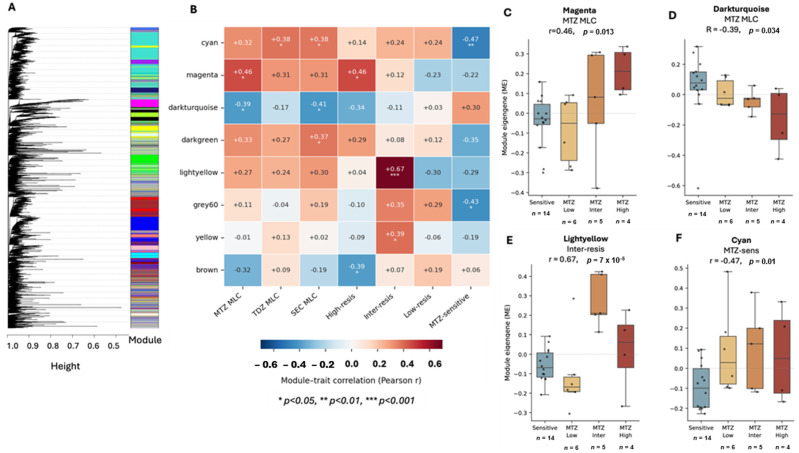
WGCNA of MTZ-, SEC-, and TDZ-resistant strains. (**A**) Hierarchical clustering dendrogram of WGCNA modules generated from VST expression matrix (top 10,000 most variable genes; signed network; soft-thresholding power selected by scale-free topology criterion R^2^ ≥ 0.85; Methods 2.5). The colored bar below the dendrogram indicates module assignments. (**B**) Module–trait correlation heatmap showing only the eight modules with at least one statistically significant correlation against a continuous MLC trait (MTZ_MLC, TDZ_MLC, SEC_MLC) or a categorical resistance-status label (MTZ-High, MTZ-Intermediate, MTZ-Low, MTZ-sensitive). Each cell shows the Pearson correlation coefficient and is shaded by direction (red = positive, blue = negative). (**C**–**F**) Module eigengene values by resistance group for the four MLC-trait-significant modules (magenta, dark turquoise, light yellow, cyan). Each box shows one module with the module name, headline trait, and correlation values as the title. Box centers are group medians, boxes are interquartile ranges, whiskers extend to the data range; individual isolates are overlaid as black points (*n* = 14 Sensitive, 6 MTZ-Low, 5 MTZ-Intermediate, 4 MTZ-High; n values listed under each *x*-axis label).

A curated 29-gene iron transport set with seven mechanistic subcategories was analyzed at the set, subcategory, and individual gene levels (Figure 4 and Figure S6). In the ZIP/SLC39 iron import subcategory (eight genes), five showed negative per-gene mean log2FC across the five comparisons, with a subcategory-level pooled mean of −0.21 (Wilcoxon signed-rank *p* = 0.058; Figure 5B). At the per-isolate level, the ZIP iron import composite score (mean z-score across the eight genes) was significantly anti-correlated with SEC-MLC (Pearson’s correlation coefficient r = −0.043, *p* = 0.02). The ferritin iron storage and cation efflux were not significantly different from the sensitive group, suggesting that the iron restriction signal is located at the entry point (Figure 4A). Multivariate analysis and elastic-net logistic regression identified the ZIP-family iron import gene *TvZIP4* (TVAG_273550) as the strongest single predictor of resistance. *TvZIP4* was selected in 98 of 100 bootstrap stability selection resamples (rank #1 of 463 candidate features) and carried the largest absolute elastic-net coefficient in the best-performing panel (AUC = 0.795, Figures S7 and S8). At the per-isolate level, *TvZIP4* expression was significantly lower in resistant versus sensitive isolates (Mann–Whitney U *p* = 0.0008 for the pooled-resistant comparison; individually significant against the MTZ-Low group *p* = 0.015 and the MTZ-Intermediate group *p* = 0.003; Figure 4C). *TvZIP4* expression was strongly anti-correlated at the per-isolate level with the compensatory iron–sulfur cluster (ISC) assembly and hydrogenosomal protein-import machinery and with Pearson’s correlation coefficient r down to −0.74 against the *DnaJ/Pam18* candidate TVAG_332890, indicating that iron import restriction and the compensatory transcriptional response co-vary at the per-isolate level rather than only at the group level (Figure 4D).

### 3.4. Weighted Gene Co-Expression Network Analysis (WGCNA) Results

WGCNA was performed on the rRNA-filtered VST expression matrix. Module–trait correlation analysis identified eight modules significantly correlated with at least one resistance trait at *p* < 0.05 (Figure 5; Tables S6 and S7). Four modules were significantly correlated with continuous MLC traits: MEmagenta (MTZ MLC r = +0.46, *p* = 0.013), MEdarkturquoise (MTZ MLC r = −0.39, *p* = 0.034; SEC MLC r = −0.41, *p* = 0.029), MEcyan (TDZ MLC *p* = 0.041; SEC MLC *p* = 0.045; MTZ-sensitive label *p* = 0.010), and MEdarkgreen (SEC r = +0.37, *p* = 0.046). Three additional modules (MEgrey60, MEbrown, MEdarkgreen) were significant only on binary resistance-status labels. All seven significant modules achieved a module preservation Z-score > 10 under both resampling analyses, indicating strong preservation of gene co-expression. The smallest module, dark turquoise, had a good Z-summary score (14.4), but low best-Jaccard overlap (0.05), indicating good co-expression, but modest sensitivity to cohort composition (Figure S1).

#### 3.4.1. Modules Associated with MTZ Resistance

The magenta module was the strongest MTZ-associated module, positively correlated with MTZ MLC (r = +0.46, *p* = 0.013). It comprises 265 genes with 50 hubs at kME ≥ 0.8 (Table S7). The dark turquoise module was negatively correlated with MTZ MLC (r = −0.39, *p* = 0.034) and contained 40 highly intercorrelated members; its hub genes were predominantly conserved hypothetical proteins (Table S7). The grey60 module was trait-significant only on the binary MTZ-sensitive label (*p* = 0.020) rather than on continuous MTZ MLC and contains the heat shock protein 70 paralog TVAG_151620 (HSP70-4) as its top hub (kME = 0.913, Table S7). This locus and a second HSP70-4 paralog (TVAG_301120) were among the few FDR-significant DEGs in the MTZ-Intermediate contrast (Section 3.2.2), linking the WGCNA modular signal directly to the protein-import compensatory machinery. The light yellow module produced the single strongest module–trait correlation in the dataset (intermediate-resistance label r = +0.67, *p* = 7 × 10^−5^; 98 genes), indicating a transcriptional program specific to the intermediate-MLC state (Table S7).

#### 3.4.2. Modules Associated with TDZ and SEC Resistance

The cyan module was significantly correlated with both TDZ MLC (r = +0.38, *p* = 0.041) and SEC MLC (r = +0.38, *p* = 0.045), and inversely with MTZ-sensitive status (r = −0.47, *p* = 0.010), making it the most cross-drug-relevant trait-significant module in the analysis (211 genes; Table S7). The dark green module was significantly correlated with SEC MLC (r = +0.37, *p* = 0.046; 54 genes). The dark turquoise module also reached significance on SEC MLC (r = −0.41, *p* = 0.029), reinforcing the iron-uptake link.

#### 3.4.3. Integration of WGCNA Modules with DEG Lists

The MTZ-MLC significant magenta module overlaps with the upregulated DEG signal in MTZ-Intermediate and MTZ-High contrasts, consistent with the predominantly upregulated DEG balance observed. The dark turquoise module, which negatively correlated with both MTZ and SEC MLCs, captured the downregulated component of the response. The grey60 module, although only binary-label trait-significant, houses the two HSP70-4 paralogs that drive the protein-import (PAM motor) DEG signal at the gene level. DGCA identified 16 candidate gene-pair correlations from the curated iron/redox list as significantly wired between sensitive and resistant isolates at FDR < 0.05. These were mostly metabolic dehydrogenases and metalloprotease members. The WGCNA module, magenta, shows the largest shift between conditions (*p* = 0.068) (Figure S5).

### 3.5. Transcript–Protein Discordance: Coordinated Iron/Redox Pathway Response

**Figure 6:**
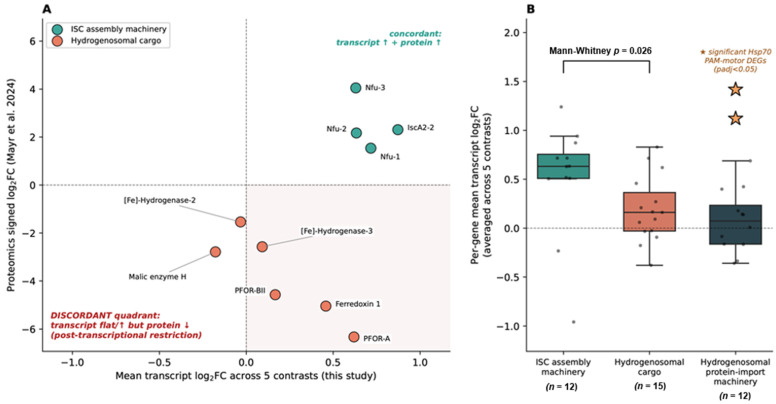
Transcript–protein discordance. (**A**) Transcript-versus-protein scatter for the 10 genes in the curated 88-gene Option B iron/redox set that had paired data in both this transcriptomic study and the Mayr et al. 2024 proteomic dataset [14]. The *x*-axis is the per-gene mean transcript log_2_ fold change across the five DESeq2 resistance contrasts; the *y*-axis is the signed log2 fold change of the protein direction reported by Mayr et al. [14]. Each point is one gene, colored by candidate group: ISC assembly machinery (teal) or hydrogenosomal cargo (orange). The upper-right quadrant (concordant up: transcript ↑ + protein ↑) contains the ISC scaffold/maturation factor genes (Nfu-1/2/3, IscA2-2). The shaded lower-right quadrant (DISCORDANT: transcript flat/↑ but protein ↓) contains the cargo genes (PFOR-A, PFOR-BII, Ferredoxin 1, [Fe]-hydrogenase-2/-3, malic enzyme H). (**B**) Per-gene mean transcript log2 fold change distribution (averaged across all five contrasts; one independent value per gene) for three candidate groups: ISC assembly machinery (*n* = 12), hydrogenosomal cargo (n = 15), and hydrogenosomal protein-import machinery (*n* = 12). Boxplots show median, interquartile range, and whiskers; black points are individual genes. The bracket annotation reports the Mann–Whitney U test comparing the ISC machinery and cargo distributions (*p* = 0.026). Orange stars on the hydrogenosomal protein-import machinery column indicate the two HSP70-4 paralogs (TVAG_151620, padj = 0.023; TVAG_301120, padj = 0.010) that reached FDR-significance as individual DEGs in the MTZ-Intermediate contrast (Methods 2.4). Proteomics direction is from Mayr et al. 2024 [14]; significance test on transcript distributions uses uncorrected Mann–Whitney U on per-gene mean log_2_FC values. ISC, iron–sulfur cluster; HSP70, heat shock protein 70.

To test whether transcriptional changes are concordant with protein-level changes from the comparative proteomics of Mayr et al. 2024 [14], a direction-stratified analysis was performed using the curated 88-gene subset of iron/redox-related genes with concordant protein-level direction across two independent mass spectrometry runs (Figure 6).

For genes annotated to the iron–sulfur cluster (ISC) assembly machinery, transcript and protein directions were concordantly upregulated in resistance: four of four ISC scaffold/maturation factor genes with paired proteomic data (Nfu-1 [TVAG_044500], Nfu-2 [TVAG_008840], Nfu-3 [TVAG_451860], IscA2-2 [TVAG_055320]) showed transcript mean log2FC > +0.6 across our five contrasts and Mayr proteomic upregulation. In contrast, for genes annotated to the hydrogenosomal cargo (PFOR paralogs, ferredoxins, [Fe]-hydrogenases, malic enzymes), six of six paired genes showed Mayr proteomic downregulation while their transcripts remained flat or only slightly elevated in our data: PFOR-A (TVAG_198110), PFOR-BII (TVAG_242960), Ferredoxin 1 (TVAG_003900), [Fe]-Hydrogenase-2 (TVAG_037570), [Fe]-Hydrogenase-3/TvhydB (TVAG_310050), and malic enzyme H (TVAG_340290), with transcript mean log2FC ranging from −0.18 to +0.62 (Figure 6).

At the group level, per-gene mean transcript log2 fold change differed significantly between the ISC machinery group (*n* = 12) and the cargo group (*n* = 15) under a Mann–Whitney U test (*p* = 0.026), with the ISC machinery skewed upward (median per-gene mean log2FC +0.63) and the cargo group near zero (median +0.16) despite the cargo group being uniformly downregulated at the protein level. This asymmetric concordance pattern, concordant transcript-up + protein-up for the assembly machinery, but discordant transcript-flat + protein-down for the cargo, is biologically consistent with post-transcriptional restriction. The assembly and import machinery are upregulated transcriptionally, but the cargo proteins (which require iron–sulfur clusters for stability) accumulate as apo-form and are cleared post-translationally, yielding protein-level depletion despite normal-to-elevated transcript levels.

Two heat shock protein 70 (HSP70-4) paralogs (TVAG_151620 padj = 0.023; TVAG_301120 padj = 0.010) reached FDR-significance in the MTZ-Intermediate transcriptomic contrast (Section 3.2.2). Both are members of the grey60 WGCNA module (Section 3.4.1), and HSP70 is a core component of the presequence-translocase-associated motor (PAM) that drives apo-protein import into the hydrogenosome. Their transcriptional upregulation provides direct evidence for activation of the protein-import compensatory pathway, the upstream half of the transcript–protein discordance pattern.

### 3.6. Cross-Comparison with Proteomics Data

A comparison of our transcriptomic data with the proteomics data of Mayr et al. [14] using the 88-gene curated subset revealed partial concordance between transcriptomic and proteomic findings for MTZ-sensitive vs. MTZ-resistance strains (Figure 6A). Several key genes involved in redox metabolism and hydrogenosomal functions, including ferredoxin, PFOR, and succinyl-CoA synthetase (SCS-α), showed consistent downregulation at the proteomic level. As detailed in Section 3.5, the transcript-level pattern for these cargo genes was systematically discordant with their proteomic direction (transcripts flat to mildly upregulated; proteins consistently downregulated), supporting a post-transcriptional restriction model. In contrast, ISC assembly/maturation factor genes (Nfu paralogs, IscA2-2) showed concordant upregulation across both platforms, with both transcript log2FC > +0.6 in our data and matched upregulation in the Mayr proteomic dataset.

Focusing on iron metabolism and redox pathways, set-level analysis showed widespread proteomic downregulation of oxidoreductases, ferredoxin-linked enzymes, and hydrogenosomal components in resistant isolates (Figures S2–S4). In our updated transcriptomic data, the iron-uptake step (Section 3.3) and cargo-protein direction (Section 3.5) match the Mayr proteomic findings under direction-stratified analysis, while the ISC assembly machinery transcriptomic signal is concordant with the proteomic data (both upregulated). Thioredoxin and peroxidase systems showed mixed but generally reduced expression at the proteomic level. The 88-gene curated set is provided in the Supplemental Files (Figure S3).

Multi-omics intersection analysis (Figure S9) identified an overlap of specific genes/proteins shared between the WGCNA hub gene set, our transcriptomic DEGs, and the Mayr proteomic dataset [14]. Notable shared loci include TVAG_160930 (periplasmic [Fe] hydrogenase), TVAG_237140 (heat shock protein 70 family member), TVAG_517010 (NADPH dehydrogenase/FR1), and several conserved hypothetical proteins. The reduced overlap size relative to earlier preliminary analyses reflects the tighter QC-screened DEG list (Section 3.2) and the curated 88-gene proteomic subset; full intersection listings are provided in the Supplemental Materials.

## 4. Discussion

Resistance to 5-nitroimidazoles in *T. vaginalis* remains a major clinical challenge because these agents constitute the only drug class approved for treatment. Previous studies have linked 5-nitroimidazole resistance to altered hydrogenosomal metabolism, impaired drug activation, and shifts in cellular redox balance [13,17]. Similarly, our previous study investigating the transcriptomic profiles of eight *T. vaginalis* isolates (four MTZ-R and four MTZ-S) also identified 304 DEGs with altered expression for important biological pathways involved in drug activation mechanisms (reduced expression) and energy metabolism [13]. The present study expands upon these findings by integrating transcriptomic profiling, co-expression network analysis, elastic-net modeling, and comparative proteomics to identify coordinated iron restriction and hydrogenosomal stress-response pathways associated with multidrug resistance.

A major finding of this study was the extensive cross-resistance observed among MTZ, TDZ, and SEC. Most MTZ-resistant isolates were also resistant to TDZ and SEC, suggesting that resistance mechanisms are largely conserved across the 5-nitroimidazole class. This observation is consistent with earlier work demonstrating that resistance in *T. vaginalis* frequently involves broad metabolic adaptations rather than drug-specific mechanisms alone [17]. The predominance of shared transcriptional signatures across resistant phenotypes in our study further supports the existence of conserved multidrug resistance pathways.

Transcriptomic profiling revealed progressively altered gene expression across increasing MTZ resistance phenotypes, with the intermediate-resistance group displaying the largest number of differentially expressed genes (DEGs). This finding is counterintuitive since we would have expected the high-resistance group to exhibit a more robust DEG profile, but in this case, it is likely due to the lower number of biological replicates, making it harder to reach an FDR of <0.05. Across all resistance contrasts, the overwhelming predominance of upregulated DEGs indicates that resistance is driven primarily through activation of adaptive transcriptional pathways rather than generalized transcriptional repression.

**Figure 7:**
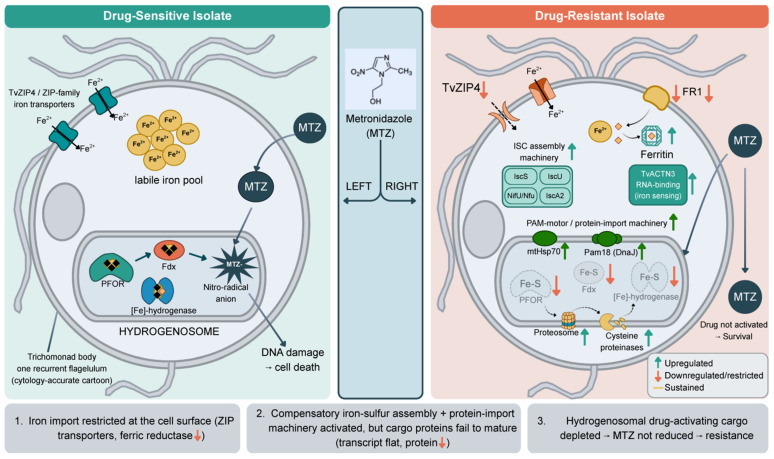
Model of potential 5-nitroimidazole resistance mechanisms. Left panel: 5-nitroimidazole-sensitive isolate showing normal iron uptake through TvZIP4/ZIP-family transporters maintaining intracellular iron availability and supporting maturation of hydrogenosomal iron–sulfur (Fe-S) cluster-containing enzymes, including pyruvate oxidoreductase (PFOR), ferredoxin (Fdx), and [Fe]-hydrogenase. These enzymes reduce metronidazole (MTZ) to cytotoxic nitro-radical intermediates, resulting in DNA damage and parasite death. Right panel: 5-nitroimidazole-resistant isolate showing reduced TvZIP4-mediated iron import and decreased flavin reductase (FR1) expression, restricting intracellular iron availability. Upregulation of iron–sulfur cluster (ISC) assembly machinery, iron-responsive RNA-binding pathways, and hydrogenosomal protein-import components (mtHsp70/Pam18) occurs. Hydrogenosomal cargo proteins fail to properly mature due to insufficient Fe incorporation and undergo proteasomal and cysteine-proteinase-mediated degradation. Loss of functional hydrogenosomal drug-activating enzymes prevents MTZ reduction, allowing the drug to remain inactive and promoting parasite survival. Bottom panels 1–3 summarize the proposed resistance model linking iron restriction and stress responses.

One of the strongest findings in this study was the identification of iron import restriction as a central feature of resistance. Iron is essential for hydrogenosomal metabolism because iron–sulfur clusters function as cofactors for pyruvate:ferredoxin oxidoreductase (PFOR), ferredoxins, and hydrogenases involved in 5-nitroimidazole activation. Previous studies have demonstrated that iron availability substantially alters hydrogenosomal protein composition and metabolic activity in *T. vaginalis* [18,19]. Our results extend these observations by showing coordinated downregulation of ZIP-family iron-uptake genes, particularly *TvZIP4*, which emerged as the strongest predictor of resistance in elastic-net modeling. Reduced *TvZIP4* expression significantly correlated with increasing resistance and inversely correlated with compensatory iron–sulfur cluster (ISC) assembly and protein-import pathways. Together, these findings support a model in which resistant isolates restrict iron uptake at the cell-membrane level, thereby limiting intracellular iron availability and reducing nitroimidazole activation within the hydrogenosome (Figure 7) [20,21,22,23,24,25,26].

The transcriptomic and proteomic analyses also provide further mechanistic insight into how resistant isolates adapt to iron limitation. Hydrogenosomes are mitochondrion-related organelles that house key iron-dependent metabolic pathways required for anaerobic energy metabolism and drug activation [27,28]. Consistent with this biology, ISC assembly and maturation genes, including *Nfu* and *IscA* paralogs, were transcriptionally upregulated in resistant isolates and showed concordant increases at the proteomic level in this study. In contrast, classical hydrogenosomal cargo proteins such as PFORs, ferredoxins, hydrogenases, and malic enzymes displayed marked transcript–protein discordance, with relatively stable transcript levels despite substantial protein depletion. This asymmetric pattern strongly suggests post-transcriptional restriction, in which insufficient iron availability impairs maturation and stabilization of iron–sulfur cluster-containing proteins, leading to degradation of apo-proteins despite preserved transcription.

These findings align with prior biochemical studies demonstrating that hydrogenosomal metabolism is highly dependent on iron availability and that iron depletion alters the abundance of hydrogenosomal oxidoreductases and electron-transfer proteins [18,19]. Our results extend those observations by suggesting that iron restriction is not simply a downstream consequence of resistance but may represent an upstream adaptive mechanism coordinating broader transcriptional and proteomic remodeling (Figure 7). Importantly, the discordance observed between transcript and protein abundance highlights the limitations of transcriptomic analyses alone for understanding 5-nitroimidazole resistance and underscores the importance of integrated multi-omics approaches.

The observed upregulation of HSP70-4 paralogs further supports activation of compensatory hydrogenosomal stress-response pathways. Hydrogenosomal proteins are synthesized in the cytosol and imported post-translationally through specialized import machinery analogous to mitochondrial systems [29,30,31]. HSP70 proteins are central components of the presequence-translocase-associated motor (PAM) that drives import of unfolded proteins into the organelle. The identification of HSP70-4 paralogs both as significant DEGs and as hub genes within the grey60 WGCNA module therefore suggests activation of a compensatory protein-import response in resistant isolates. Increased expression of protein-import machinery may reflect an attempt to maintain hydrogenosomal homeostasis despite impaired maturation and turnover of iron-dependent cargo proteins.

WGCNA further demonstrated that resistance is organized at the pathway and systems level rather than through isolated gene-level changes. Several modules correlated significantly with MTZ, TDZ, and SEC resistance phenotypes, including modules enriched for adaptive stress-response genes and negatively correlated iron-uptake genes. Notably, the light yellow module exhibited the strongest association with intermediate-resistance status, further supporting the hypothesis that intermediate resistance represents a distinct transitional transcriptional state. The cyan module, which correlated with both TDZ and SEC resistance while inversely correlating with MTZ-sensitive status, likely represents a conserved multidrug resistance network shared across the 5-nitroimidazole class.

Our findings are broadly consistent with earlier models proposing that 5-nitroimidazole resistance arises through reduced drug activation within the hydrogenosome [19]. However, the present study extends previous work by identifying iron import restriction and compensatory ISC assembly activation as potentially upstream regulatory events linking hydrogenosomal dysfunction, altered redox metabolism, and multidrug resistance. The strong predictive performance and stability of *TvZIP4* further identify this locus as a promising candidate biomarker for resistance surveillance and future functional validation studies.

Several limitations of this study should be acknowledged. First, it was done in vitro and may not translate to in vivo clinical conditions. Additionally, this study itself did not include a proteomic analysis of the specific *T. vaginalis* isolates utilized in this research. The benefit of combining a proteomics approach with transcriptomic data would provide a more comprehensive, multi-layered view of the biological systems involved with the tested traits of low, medium, and high MLCs for MTZ-resistant isolates. This would enable us to better understand and connect gene expression directly to the translation of functional proteins, considering the effects of post-transcriptional regulation, translation modulation, and degradation. The sample size was also modest, particularly within individual resistance subgroups, which may have limited statistical power for the detection of smaller transcriptional effects. The substantial heterogeneity observed among TDZ-resistant isolates likely contributed to the relatively small number of significant DEGs in that comparison. Additionally, while the integrated transcriptomic and proteomic analyses support a mechanistic model of iron restriction-mediated resistance, direct functional studies will be necessary to establish causality and define the specific contribution of *TvZIP4*, ISC assembly pathways, and hydrogenosomal protein-import machinery to resistance development.

In conclusion, this study demonstrates that 5-nitroimidazole resistance in *T. vaginalis* is associated with coordinated transcriptional and post-transcriptional remodeling centered on iron restriction, hydrogenosomal stress adaptation, and compensatory ISC assembly activation. Integration with prior hydrogenosomal and iron-metabolism studies supports a model in which altered iron homeostasis acts as a central upstream regulator of multidrug resistance. These findings provide a broader mechanistic framework for understanding 5-nitroimidazole resistance in *T. vaginalis* and identifying candidate biomarkers and pathways that may support future therapeutic and diagnostic development.

## Figures and Tables

**Table 1:** Characterization of 5-nitroimidazole minimum lethal concentrations (MLCs) (µg/mL) among clinical *T. vaginalis* isolates.

STATUS	ID	MTZ MLC	TDZ MLC	SEC MLC
**HIGH MLC RESISTANCE** MTZ MLC ≥ 400 µg/mL	CDC0685	400	400	50
	CDC1631	400	50	3.1
	CDC6505	400	400	25
	TV009	400	25	100
**INTERMEDIATE MLC RESISTANCE** MTZ MLC = 100–200 µg/mL	CDC1629	200	25	200
	CDC1650	200	25	3.1
	CDC5601	100	50	3.1
	LUP009	100	50	50
	VC004	100	12.5	50
**LOW MLC RESISTANCE** MTZ MLC = 50 µg/mL	CDC1550	50	6.3	50
	CDC1582	50	3.1	1.6
	TV1062	50	0.8	25
	VC005	50	100	25
	VC006	50	25	50
	VC010	50	12.5	50
**MTZ-SENSITIVE MLCs** MTZ MLC < 50 µg/mL	TV1054	25	0.8	6.3
	LUP007	12.5	3.1	1.6
	WF001	12.5	1.6	12.5
	LUP005	6.3	3.1	3.1
	LUP006	6.3	1.6	12.5
	CDC520	3.1	0.8	1.6
	LUP001	3.1	0.8	6.3
	LUP002	3.1	1.6	0.2
	LUP004	3.1	0.8	1.6
	VC001	3.1	1.6	3.1
	ECU001	1.6	3.1	3.1
	LUP003	1.6	1.6	12.5
	LUP008	1.6	1.6	0.8
	TV1038	0.4	1.6	≤0.2
	VC003	0.4	0.8	1.6

Abbreviations: ID = identification number, MTZ = metronidazole, TDZ = tinidazole, SEC = secnidazole, MLC = minimal lethal concentration.

## Data Availability

The original data presented in the study are openly available in the NCBI Gene Expression Omnibus (GEO) under accession number GSE326939.

## Supplementary Materials

The following supporting information can be downloaded at https://www.mdpi.com/article/10.3390/microorganisms14061314/s1: Table S1. MTZ-Low vs. MTZ-sensitive—significant DEGs; Table S2. MTZ-Intermediate vs. MTZ-sensitive—significant DEGs; Table S3. MTZ-High vs. MTZ-sensitive—significant DEGs; Table S4. TDZ-High vs. MTZ-sensitive—significant DEGs; Table S5. SEC-High vs. MTZ-sensitive—significant DEGs; Table S6. WGCNA module–trait correlations; Table S7. WGCNA hub genes for the four MLC-trait-significant modules; Figure S1: WGCNA module stability under leave-one-out and bootstrap resampling; Figure S2. Gene Set Variation Analysis (GSVA) on iron/redox gene set scoring; Figure S3. GSVA—Option B (Mayr-validated) gene set scoring; Figure S4. GSVA—Option A1 subcategory heatmap and trait correlation; Figure S5. Differential gene correlation analysis (DGCA) of T. vaginalis resistance; Figure S6. Iron transport detail panels; Figure S7. Multivariate sPLS-DA/elastic-net classifier performance; Figure S8. Multivariate sPLS-DA/elastic-net classifier, feature stability, and ordination; Figure S9. Comprehensive 56-TVAG Deep Research candidate gene canvas.
