# Supplementary material for: Transcriptomic Signatures of Trichomonas vaginalis Isolates That Exhibit Low, Intermediate, and High In Vitro Resistance to Metronidazole
Source: Microorganisms. 2026 Jun 12;14(6):1314. doi: 10.3390/microorganisms14061314 (PMC13303814; doi:10.3390/microorganisms14061314)
Supplement: Supplementary file 1 [file microorganisms-14-01314-s001.zip › microorganisms-4291912-Supplementary Figures.pdf]

Supplemental Figures

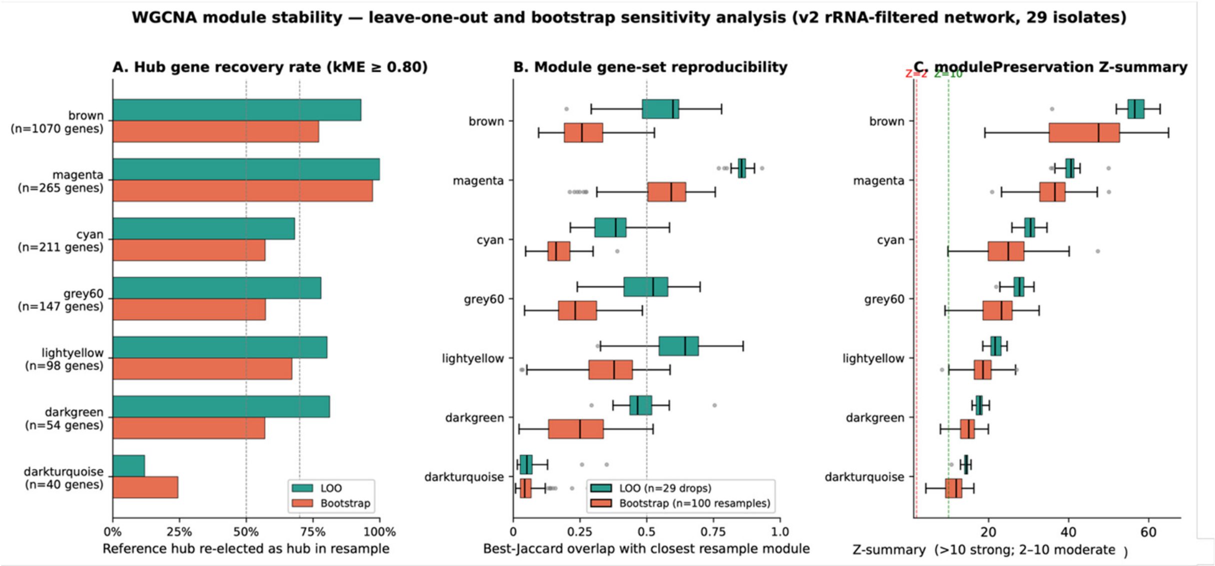

Figure S1: WGCNA module stability under leave-one-out and bootstrap resampling.

Stability of the rRNA-filtered network's trait-significant modules across 29 leave-one-out rebuilds (one isolate dropped per rebuild; green) and 100 bootstrap rebuilds (29 isolates sampled with replacement; orange). (A) Hub-gene recovery rate. Fraction of reference hubs (kME ≥ 0.80) that were re-elected as a hub of any module in the resampled network, averaged across resampling iterations. Vertical dashed lines mark 50% and 70% reference levels. (B) Best-Jaccard module overlap. For each resampling iteration, the Jaccard overlap of the reference module's gene set against the best-matching module in the resampled network. Boxplots show the median, interquartile range, and full range across iterations (LOO: n = 29 points per module; bootstrap: n = 100). Vertical dashed line at 0.5 marks the point at which a resampled module shares the majority of its genes with the reference. (C) WGCNA::modulePreservation Z-summary across resampled networks. Z-summary > 10 indicates strong preservation, 2–10 moderate, and < 2 not preserved (vertical dashed lines mark Z = 2 and Z = 10). Z-summary integrates density-based and connectivity-based preservation statistics relative to a null derived from 100 module-label permutations. The four MLC-trait-significant modules are magenta, darkturquoise, lightyellow, and cyan; the three binary-label-only modules are grey60, brown, and darkgreen. LOO, leave-one-out; kME, module membership.

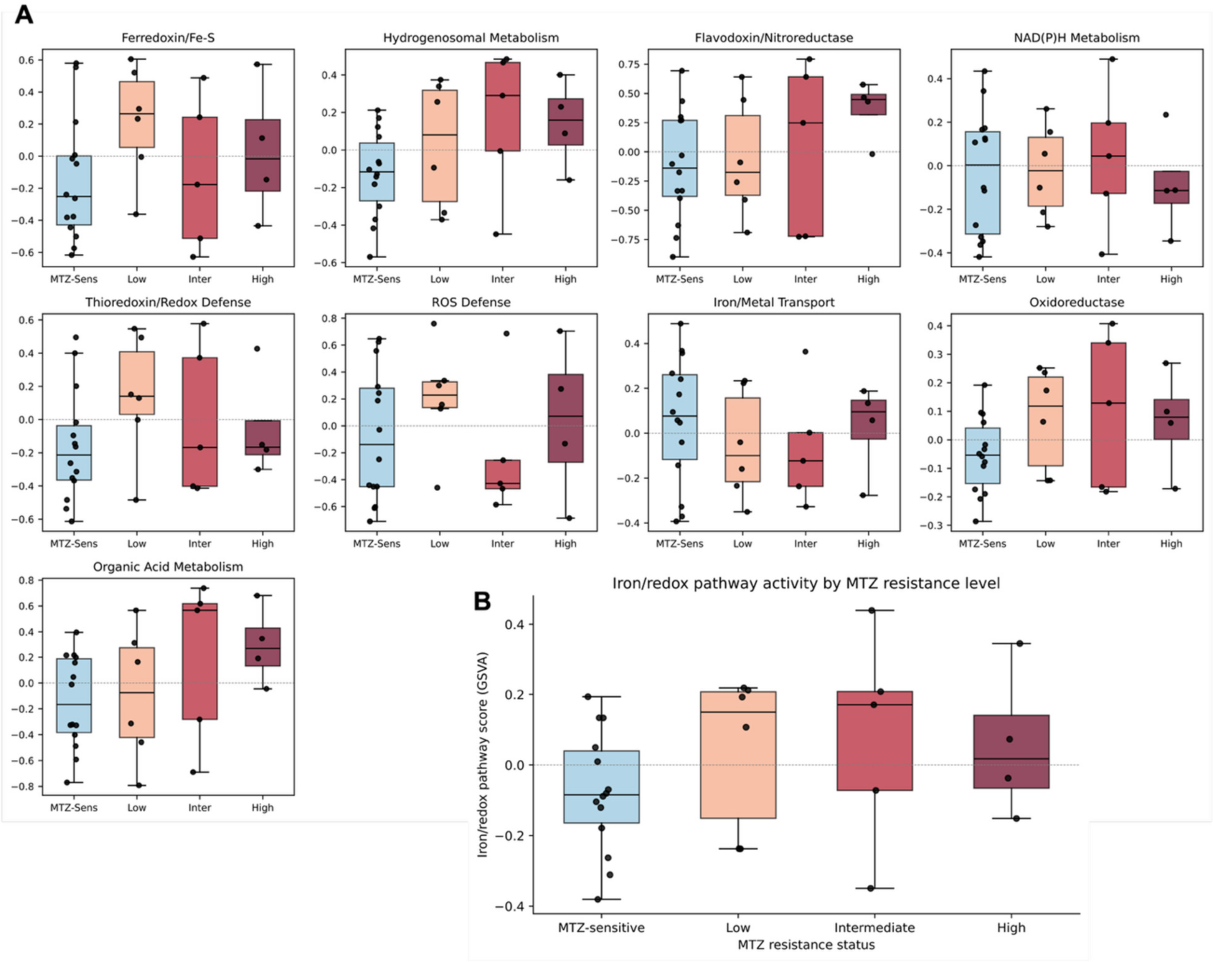

Figure S2. Gene Set Variation Analysis (GSVA) on iron/redox gene set scoring.

(A) GSVA score distribution for the Option A iron/redox gene set (*n* = 426 genes; Methods 2.6) across the four resistance-status groups, shown as boxplots. (B) Per-subcategory GSVA score boxplots for the 11 mechanistic subcategories of the Option A1 partitioning (PFOR/Ferredoxin Cycle, Ferredoxin/Fe-S, Hydrogenosomal Metabolism, Flavodoxin/Nitroreductase, NAD(P)H Metabolism, Thioredoxin/Redox Defense, ROS Defense, Iron/Metal Transport, Oxidoreductase, Organic Acid Metabolism, Cytochrome/Heme), grouped by resistance status. MLC, minimum lethal concentration; PFOR, pyruvate:ferredoxin oxidoreductase; ROS, reactive oxygen species; NAD(P)H, nicotinamide adenine dinucleotide (phosphate).

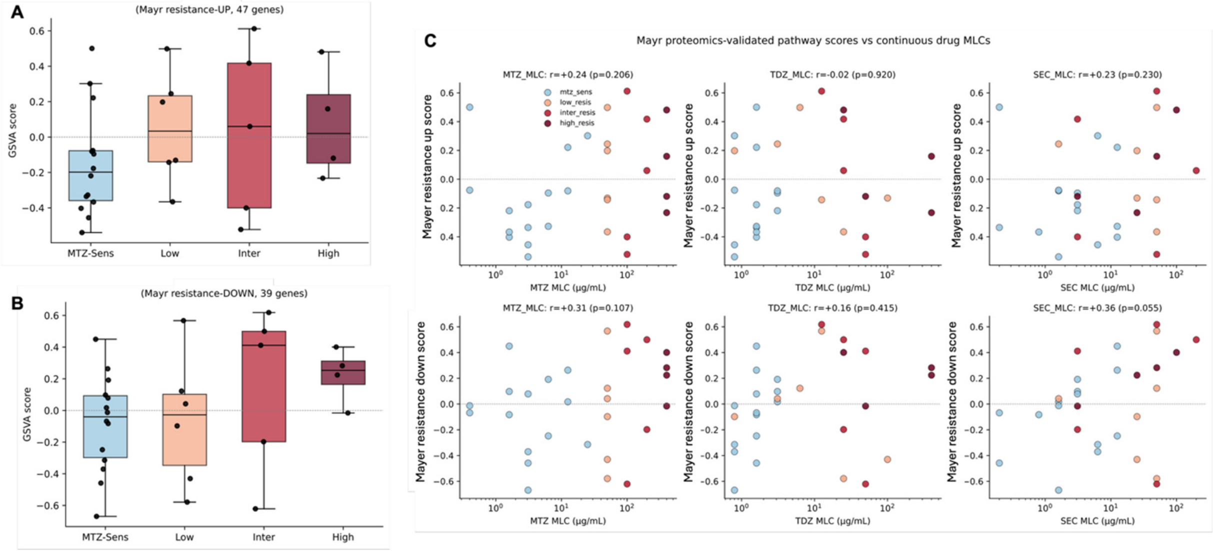

Figure S3. GSVA — Option B (Mayr-validated) gene set scoring.

(A-B) GSVA score distribution by resistance-status group for the Mayr-validated 88-gene iron/redox subset, shown as boxplots for genes up regulated and down regulated. (C) Scatter plot of per-isolate GSVA score versus $\log_2$(MTZ MLC), with the Pearson correlation coefficient and p-value reported. MLC, minimum lethal concentration.

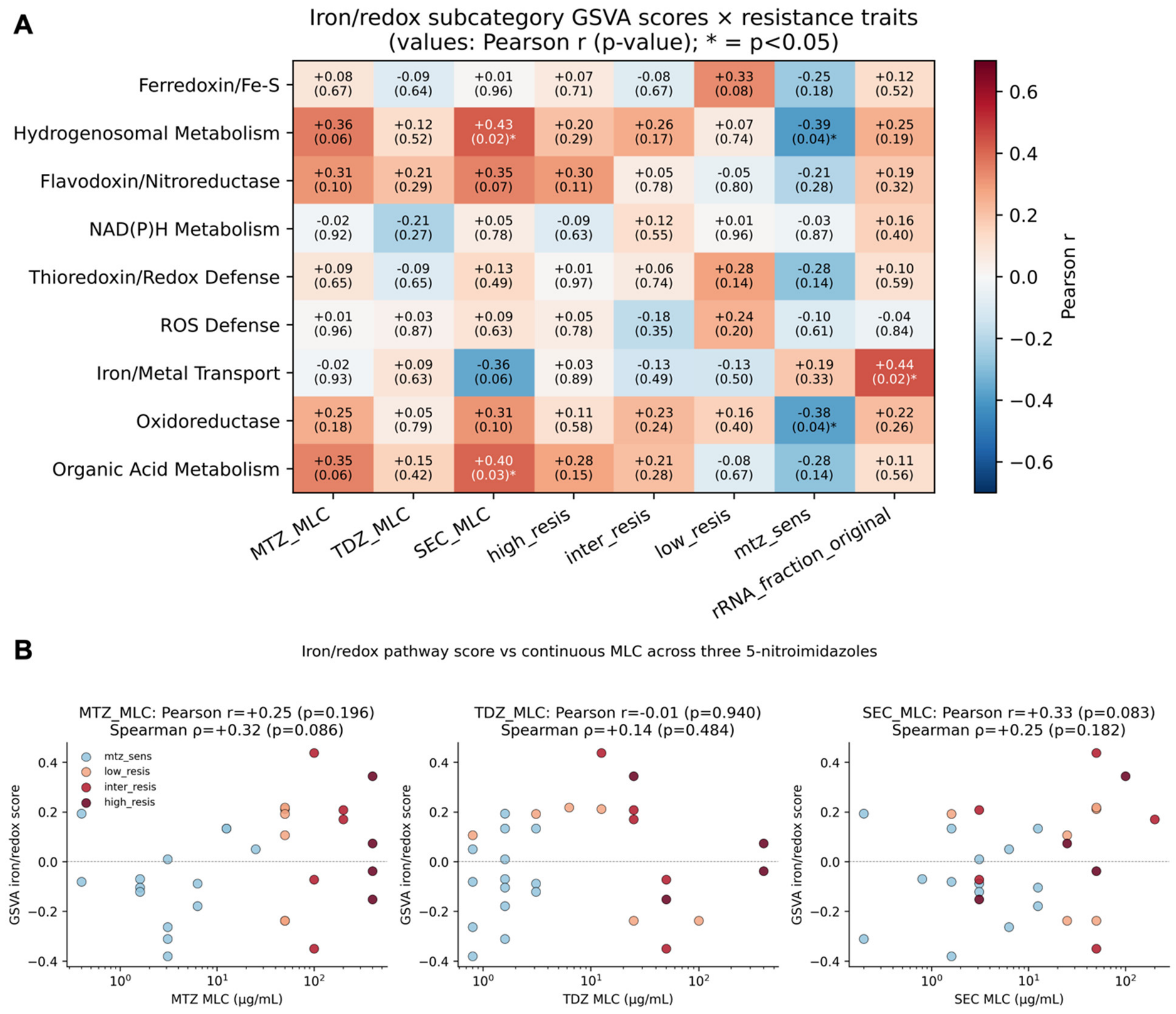

Figure S4. GSVA — Option A1 subcategory heatmap and trait correlation.

(A) Heatmap of per-isolate Option A1 subcategory GSVA scores (rows: 11 mechanistic subcategories; columns: 29 isolates ordered by ascending MTZ MLC). Color scale indicates z-scored GSVA score. (B) Scatter plot of per-isolate Option A GSVA score versus $\log_2$(MTZ MLC), with the Pearson correlation coefficient and p-value reported. MLC, minimum lethal concentration; FC, fold change.

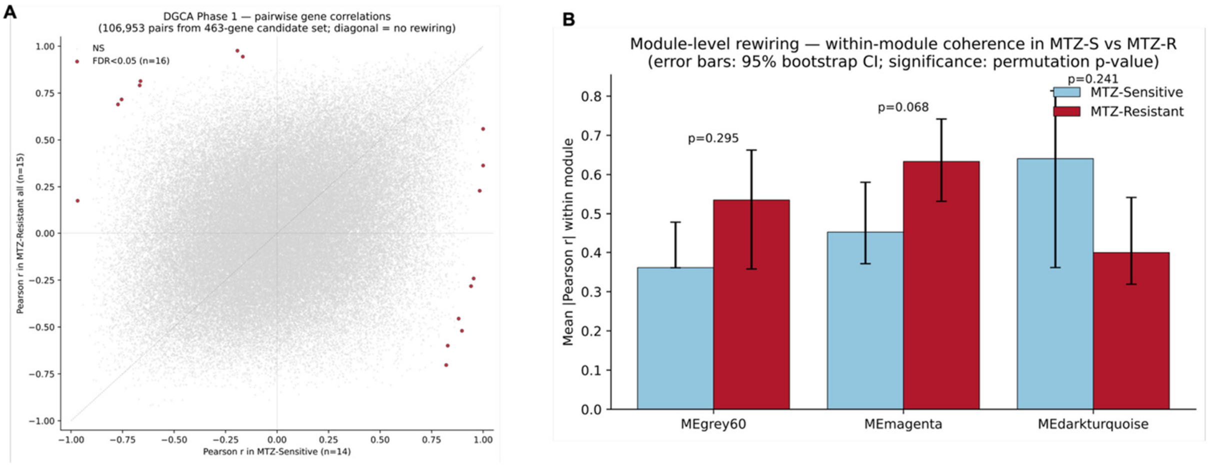

Figure S5. Differential gene correlation analysis (DGCA) of *T. vaginalis* resistance.

(A) Phase 1 differential correlation scatter showing pairwise gene–gene Pearson correlations under sensitive (x-axis) versus resistant (y-axis) conditions, for 107,253 candidate pairs from the curated iron/redox set. Points on the diagonal are not differentially correlated; off-diagonal points indicate rewired correlations. Pairs with FDR-adjusted differential correlation $p < 0.05$ are color-highlighted. (B) Phase 2 module-level rewiring summary showing the number of significantly rewired pairs within each WGCNA module.

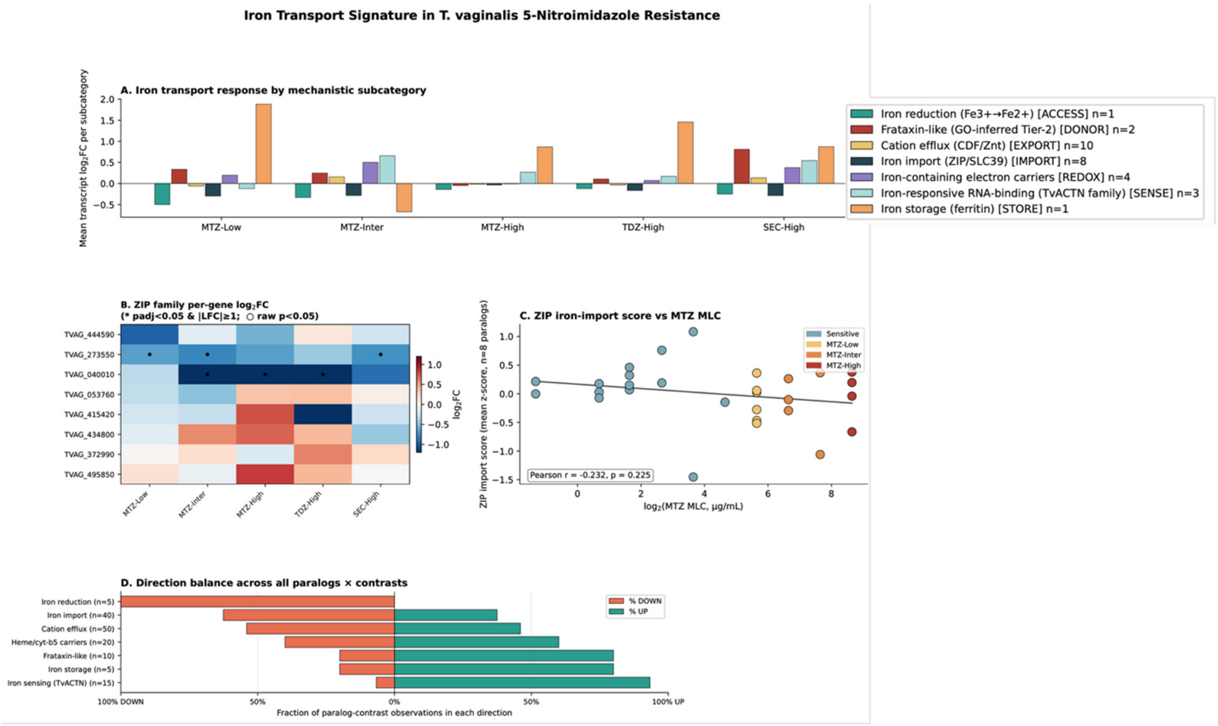

Figure S6. Iron transport detail panels

Iron transport analyses from the curated 29-gene set. (A) Iron transport restriction by mechanistic subcategory: grouped bar chart of mean transcript $\log_2$FC per subcategory across the five resistance contrasts, colored by functional direction. (B) ZIP family (n = 8 paralogs) per-gene $\log_2$FC heatmap across the five resistance contrasts. Each row is a single ZIP paralog labeled by TVAG locus ID; cell color shows $\log_2$FC. Asterisks (*) mark cells with padj < 0.05 and $|\log_2 FC| \geq 1$; open circles (○) mark cells with raw $p < 0.05$ only. (C) ZIP iron-import score versus MTZ MLC, the Pearson r and p-value are reported as an in-panel annotation. (D) Direction balance by subcategory: stacked horizontal bar chart showing, for each of the seven mechanistic subcategories (Iron import [ZIP/SLC39], Iron reduction [$Fe^{3+} \rightarrow Fe^{2+}$], Cation efflux [CDF/Znt], Iron-containing electron carriers, Iron storage [ferritin], Iron-responsive RNA-binding [TvACTN family], Frataxin-like [Tier-2]), the fraction of paralog × contrast observations going in each direction (red = down, teal = up), with the total number of observations annotated. Subcategories are ordered by ascending fraction-DOWN. ZIP, Zrt-/Irt-like Protein; SLC39, solute carrier family 39; CDF, cation diffusion facilitator; Znt, zinc transporter; FC, fold change; padj, Benjamini–Hochberg adjusted p-value.

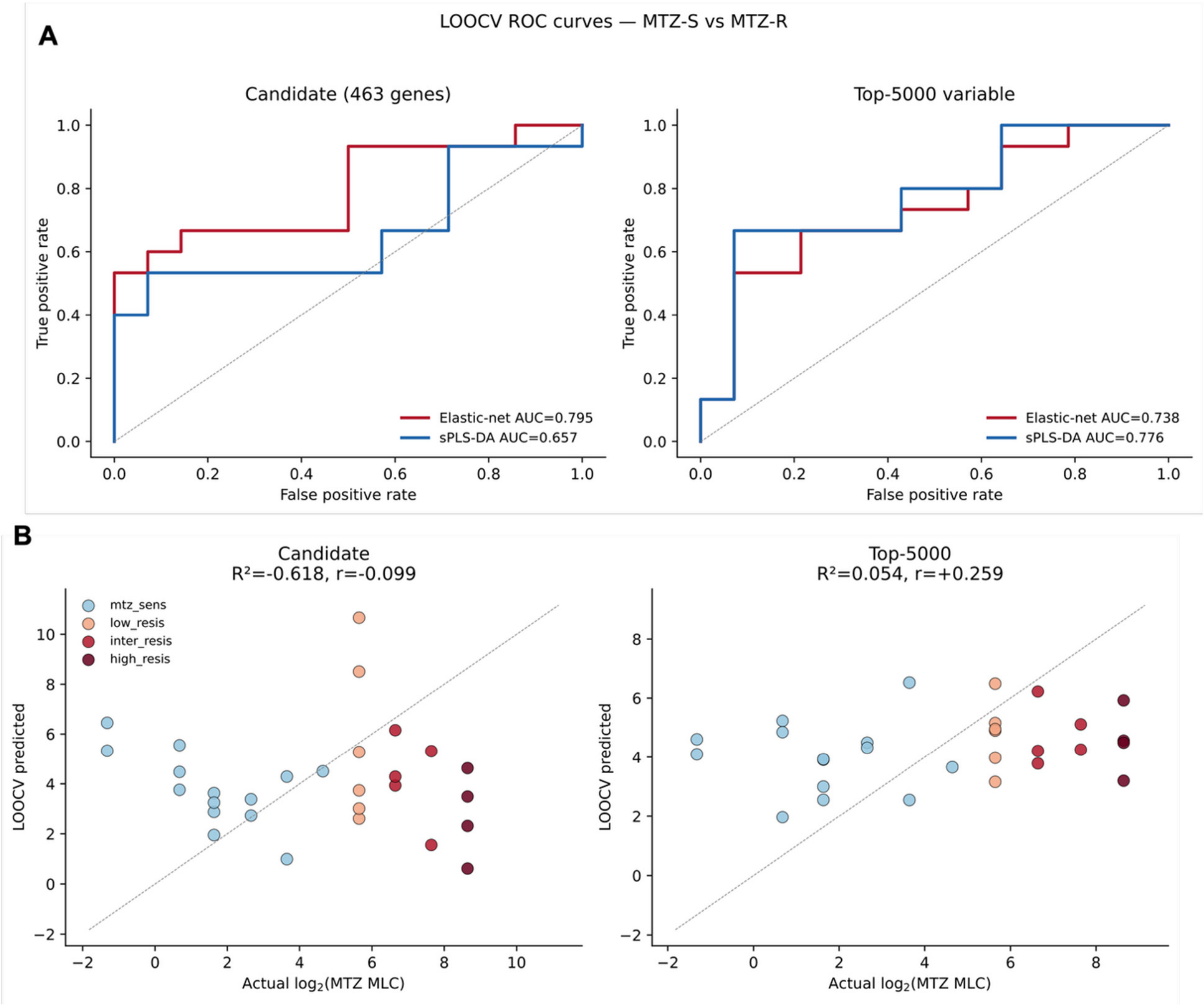

Figure S7. Multivariate sPLS-DA / elastic-net classifier performance.

(A) Receiver operating characteristic (ROC) curve for the best-performing classifier panel (candidate iron/redox gene set × binary MTZ-resistance status), trained under leave-one-out cross-validation across the 29 isolates with elastic-net regularization (Methods 2.6). Area under the curve (AUC) = 0.795. (B) Predicted $\log_2$(MTZ MLC) versus observed $\log_2$(MTZ MLC) for the continuous-outcome variant of the candidate-gene panel. Each point is one isolate; the diagonal line represents perfect prediction; the Pearson r and prediction R^2 are reported. AUC, area under the curve.

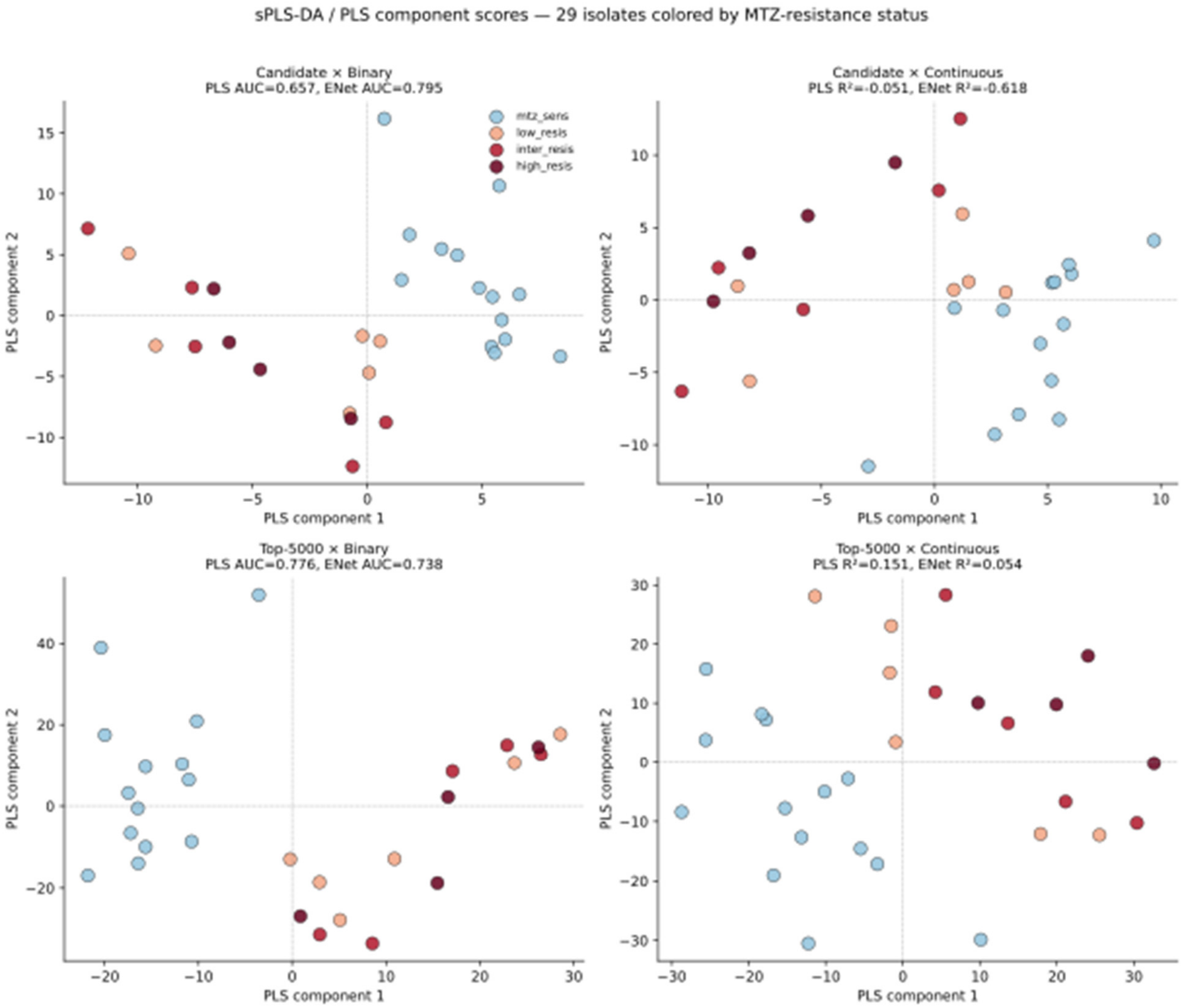

Figure S8. Multivariate sPLS-DA / elastic-net classifier, feature stability and ordination.

Sparse partial least squares discriminant analysis (sPLS-DA) score plot of the first two latent components for the 29 isolates, colored by MTZ resistance status. Separation along the first component reflects the iron/redox transcriptional signature. sPLS-DA, sparse partial least squares discriminant analysis.

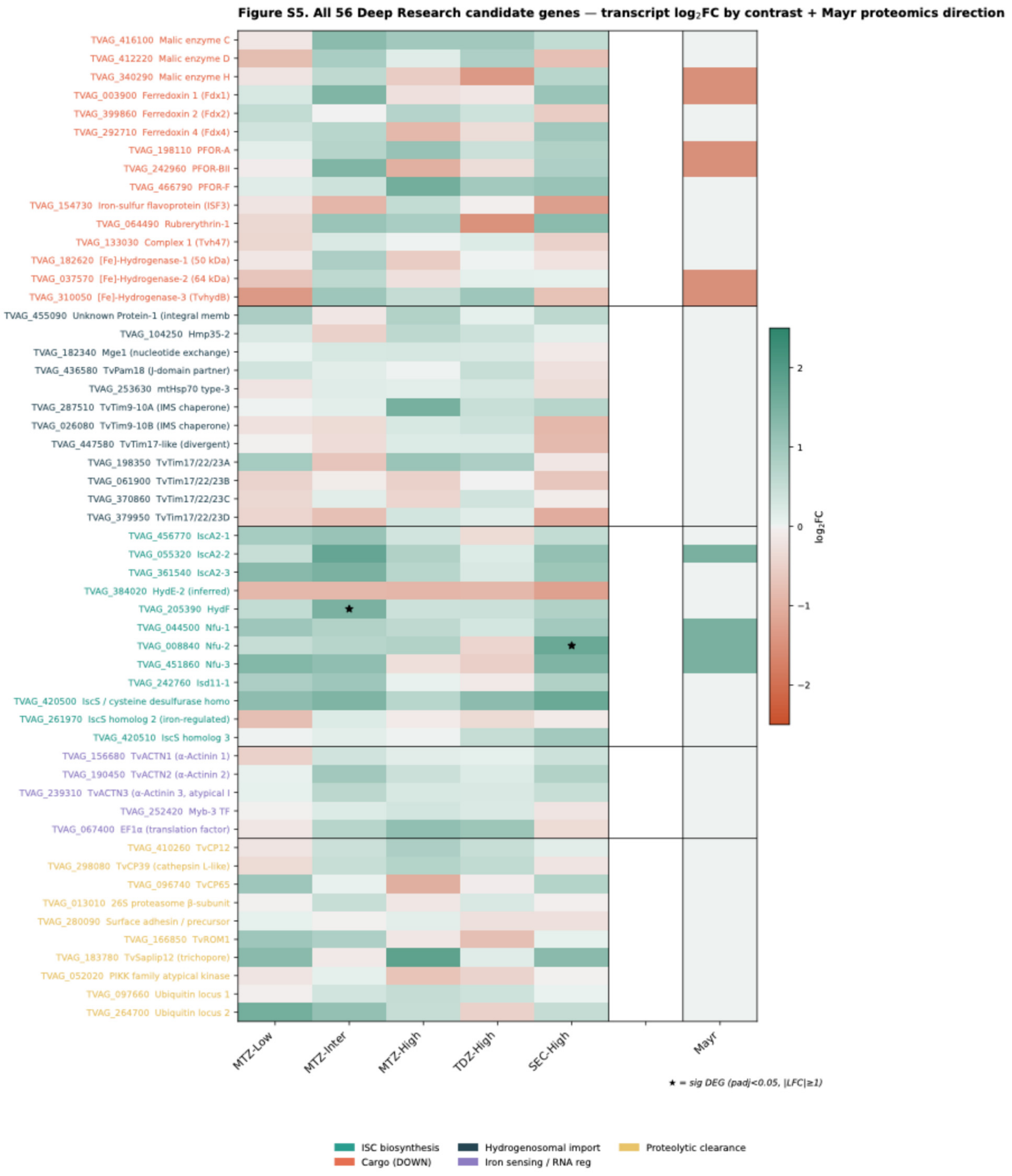

Figure S9. Comprehensive 56-TVAG Deep Research candidate gene canvas.

Heatmap of all 56 candidate TVAG locus IDs from the literature-derived Fe-S regulator candidate list (Methods 2.6) versus the five resistance contrasts (columns 1–5: MTZ-Low, MTZ-Intermediate, MTZ-High, TDZ-High, SEC-High; column 6 [right]: Mayr et al. proteomics direction).

Cell color indicates transcript log_2FC (red = upregulated, blue = downregulated, white = no change); the Mayr column uses the same colormap to indicate signed protein-level direction. Asterisks (*) mark cells with padj < 0.05 and $|log_2FC| \geq 1$. Rows are grouped and color-coded by Deep Research candidate category: ISC biosynthesis (teal), hydrogenosomal cargo (orange), hydrogenosomal protein-import machinery (dark blue), iron sensing / RNA-binding regulators (purple), and proteolytic clearance (mustard). Horizontal black lines separate categories.
