# Supplementary material for: Transcriptomic Signatures of Trichomonas vaginalis Isolates That Exhibit Low, Intermediate, and High In Vitro Resistance to Metronidazole
Source: Microorganisms. 2026 Jun 12;14(6):1314. doi: 10.3390/microorganisms14061314 (PMC13303814; doi:10.3390/microorganisms14061314)
Supplement: Supplementary file 1 [file microorganisms-14-01314-s001.zip › microorganisms-4291912-Supplementary Tables.pdf]

Supplementary Tables

Table S1. MTZ-Low vs MTZ-sensitive — significant DEGs (n = 28; 27 upregulated, 1 downregulated)

Significant differentially expressed genes (padj < 0.05 and |log2 fold change| ≥ 1) from the QC-screened DESeq2 analysis. Positive log2FC = upregulated in resistance. Ranked by padj.

Rank	TVAG_ID	Protein_ID	log2FC	p-value	padj	Direction	Product_Description
1	TVAG_371320	EAX90071	+3.12	4.67e-06	1.20e-02	Up	malate dehydrogenase, putative
2	TVAG_190330	EAY19138	+3.70	9.00e-06	1.20e-02	Up	lipid A export ATP-binding/permease protein msba, putative
3	TVAG_300820	EAX94549	+2.52	8.10e-06	1.20e-02	Up	conserved hypothetical protein
4	TVAG_483010	EAX98044	+2.56	4.35e-06	1.20e-02	Up	conserved hypothetical protein
5	TVAG_161060	EAY12300	-2.45	6.65e-06	1.20e-02	Down	ankyrin repeat-containing protein, putative
6	TVAG_147870	EAX91140	+1.01	6.23e-06	1.20e-02	Up	conserved hypothetical protein
7	TVAG_069570	EAY06587	+1.81	2.21e-06	1.20e-02	Up	ubiquitin 1, putative
8	TVAG_126230	EAY09462	+3.12	1.52e-05	1.78e-02	Up	conserved hypothetical protein
9	TVAG_021440	EAY20198	+1.78	1.95e-05	1.82e-02	Up	histone H2a, putative
10	TVAG_019660	EAY15065	+6.58	1.80e-05	1.82e-02	Up	conserved hypothetical protein
11	TVAG_482800	EAX92404	+2.82	3.84e-05	1.90e-02	Up	conserved hypothetical protein
12	TVAG_425600	EAX95375	+2.59	3.07e-05	1.90e-02	Up	alpha-amylase, putative
13	TVAG_254860	EAY02585	+1.38	3.86e-05	1.90e-02	Up	conserved hypothetical protein
14	TVAG_031780	EAY03679	+2.87	3.68e-05	1.90e-02	Up	r2r3-MYB transcription factor, putative
15	TVAG_417070	EAY04409	+2.84	2.33e-05	1.90e-02	Up	conserved hypothetical protein
16	TVAG_173120	EAY21054	+4.34	2.89e-05	1.90e-02	Up	abca9, putative

**17**	TVAG_224270	EAY15351	+1.65	3.65e-05	1.90e-02	Up	CMGC family protein kinase
**18**	TVAG_470680	EAY06225	+1.12	3.25e-05	1.90e-02	Up	histone H4, putative
**19**	TVAG_050340	EAY07219	+2.74	3.72e-05	1.90e-02	Up	conserved hypothetical protein
**20**	TVAG_399700	EAY11964	+2.44	5.45e-05	2.38e-02	Up	conserved hypothetical protein
**21**	TVAG_226780	EAX97434	+1.39	5.62e-05	2.38e-02	Up	histone H2b, putative
**22**	TVAG_493420	EAX96400	+2.13	5.86e-05	2.38e-02	Up	conserved hypothetical protein
**23**	TVAG_026380	EAY14317	+1.58	5.78e-05	2.38e-02	Up	histone H2a, putative
**24**	TVAG_161100	EAY12304	+2.27	6.35e-05	2.47e-02	Up	heat shock protein 70kD, putative
**25**	TVAG_387950	EAY13654	+2.87	6.91e-05	2.58e-02	Up	conserved hypothetical protein
**26**	TVAG_050180	EAY07203	+3.83	1.11e-04	4.00e-02	Up	modulator of drug activity B, putative
**27**	TVAG_466130	EAY02753	+2.34	1.45e-04	4.82e-02	Up	conserved hypothetical protein
**28**	TVAG_110540	EAY20442	+1.79	1.41e-04	4.82e-02	Up	ubiquitin, putative

Table S2. MTZ-Intermediate vs MTZ-sensitive — significant DEGs (n = 140; 134 upregulated, 6 downregulated)

Significant differentially expressed genes (padj < 0.05 and |log2 fold change| ≥ 1) from the QC-screened DESeq2 analysis. Positive log2FC = upregulated in resistance. Ranked by padj.

Rank	TVAG_ID	Protein_ID	log2FC	p-value	padj	Direction	Product_Description
**1**	TVAG_380290	EAY14919	+3.21	6.88e-11	1.30e-06	Up	conserved hypothetical protein
**2**	TVAG_503660	EAX78014	+3.13	3.92e-10	3.69e-06	Up	conserved hypothetical protein
**3**	TVAG_345530	EAX90993	+2.64	6.00e-10	3.77e-06	Up	conserved hypothetical protein
**4**	TVAG_415490	EAX88457	+3.06	2.69e-09	1.27e-05	Up	conserved hypothetical protein
**5**	TVAG_332890	EAY07964	+1.57	5.99e-09	2.26e-05	Up	chaperone protein DNAj, putative
**6**	TVAG_399800	EAX75545	+3.79	2.08e-08	4.89e-05	Up	conserved hypothetical protein
**7**	TVAG_132230	EAX93506	+2.63	1.98e-08	4.89e-05	Up	conserved hypothetical protein

**8**	TVAG_399490	EAY11944	+2.90	1.59e-08	4.89e-05	Up	Microtubule-associated protein futsch, putative
**9**	TVAG_062580	EAY18653	+2.78	3.37e-08	6.35e-05	Up	sphingomyelin phosphodiesterase, putative
**10**	TVAG_380280	EAY14918	+2.24	3.13e-08	6.35e-05	Up	conserved hypothetical protein
**11**	TVAG_399260	EAY11921	+2.98	7.00e-08	1.20e-04	Up	conserved hypothetical protein
**12**	TVAG_097870	EAY13217	+2.72	9.42e-08	1.48e-04	Up	conserved hypothetical protein
**13**	TVAG_233640	EAX94113	+2.87	1.41e-07	2.04e-04	Up	conserved hypothetical protein
**14**	TVAG_371320	EAX90071	+4.21	2.02e-07	2.72e-04	Up	malate dehydrogenase, putative
**15**	TVAG_306580	EAY18136	+2.19	2.52e-07	3.03e-04	Up	conserved hypothetical protein
**16**	TVAG_417060	EAY04408	+2.10	2.57e-07	3.03e-04	Up	conserved hypothetical protein
**17**	TVAG_482800	EAX92404	+3.68	3.86e-07	4.28e-04	Up	conserved hypothetical protein
**18**	TVAG_227950	EAX81953	+2.37	4.90e-07	5.13e-04	Up	conserved hypothetical protein
**19**	TVAG_554600	EAX68806	+2.65	6.25e-07	6.20e-04	Up	conserved hypothetical protein
**20**	TVAG_399750	EAX86010	+1.47	6.89e-07	6.49e-04	Up	adenylate cyclase type VI, putative
**21**	TVAG_227100	EAX96215	+1.76	8.87e-07	7.96e-04	Up	conserved hypothetical protein
**22**	TVAG_153560	EAY05322	+2.10	1.43e-06	1.22e-03	Up	heat shock protein, putative
**23**	TVAG_151660	EAY06359	+2.38	2.50e-06	2.05e-03	Up	conserved hypothetical protein
**24**	TVAG_547940	EAX79580	+2.75	3.13e-06	2.46e-03	Up	conserved hypothetical protein
**25**	TVAG_416510	EAY05008	+2.31	3.70e-06	2.60e-03	Up	adenosine deaminase, putative
**26**	TVAG_416980	EAY04400	+3.08	3.64e-06	2.60e-03	Up	conserved hypothetical protein
**27**	TVAG_466130	EAY02753	+2.76	3.79e-06	2.60e-03	Up	conserved hypothetical protein
**28**	TVAG_098470	EAY09712	+2.44	3.87e-06	2.60e-03	Up	conserved hypothetical protein
**29**	TVAG_454270	EAY17599	+1.76	4.56e-06	2.96e-03	Up	heat shock protein, putative

**30**	TVAG_161100	EAY12304	+1.73	5.93e-06	3.22e-03	Up	heat shock protein 70kD, putative
**31**	TVAG_546550	EAX74772	+4.01	5.60e-06	3.22e-03	Up	conserved hypothetical protein
**32**	TVAG_496810	EAX93359	+2.40	5.97e-06	3.22e-03	Up	conserved hypothetical protein
**33**	TVAG_434140	EAY16577	+1.48	5.88e-06	3.22e-03	Up	trichohyalin, putative
**34**	TVAG_476690	EAY22740	+1.86	6.15e-06	3.22e-03	Up	DNAj/HSP40, putative
**35**	TVAG_009460	EAX89112	+3.04	6.09e-06	3.22e-03	Up	malic enzyme, putative
**36**	TVAG_083240	EAY18471	+1.76	5.92e-06	3.22e-03	Up	DNA-binding protein reb1, putative
**37**	TVAG_232340	EAX81728	+3.16	6.51e-06	3.32e-03	Up	conserved hypothetical protein
**38**	TVAG_425600	EAX95375	+2.45	6.86e-06	3.40e-03	Up	alpha-amylase, putative
**39**	TVAG_034720	EAX95043	+1.96	8.43e-06	4.07e-03	Up	heat shock protein, putative
**40**	TVAG_139320	EAY12552	+2.28	9.56e-06	4.50e-03	Up	heat shock protein, putative
**41**	TVAG_227940	EAX81952	+2.64	9.80e-06	4.50e-03	Up	conserved hypothetical protein
**42**	TVAG_500630	EAX75018	+3.04	1.09e-05	4.91e-03	Up	conserved hypothetical protein
**43**	TVAG_380670	EAX90771	+2.13	1.17e-05	5.13e-03	Up	conserved hypothetical protein
**44**	TVAG_228560	EAY19593	+2.08	1.24e-05	5.30e-03	Up	conserved hypothetical protein
**45**	TVAG_188320	EAY15781	+2.68	1.27e-05	5.32e-03	Up	conserved hypothetical protein
**46**	TVAG_189610	EAY01175	+3.91	1.51e-05	6.20e-03	Up	conserved hypothetical protein
**47**	TVAG_109650	EAY10381	+1.73	1.55e-05	6.21e-03	Up	caldesmon, putative
**48**	TVAG_454570	EAY03818	+2.16	1.59e-05	6.26e-03	Up	Embryonic protein DC-8, putative
**49**	TVAG_228520	EAY19589	+3.26	1.72e-05	6.61e-03	Up	malic enzyme, putative
**50**	TVAG_259370	EAY09856	+2.73	1.93e-05	6.85e-03	Up	conserved hypothetical protein
**51**	TVAG_291650	EAY17213	+2.87	1.85e-05	6.85e-03	Up	conserved hypothetical protein
**52**	TVAG_319090	EAY05554	+1.57	1.86e-05	6.85e-03	Up	conserved hypothetical protein
**53**	TVAG_328180	EAX90717	+1.86	1.91e-05	6.85e-03	Up	proteasome-activating

							nucleotidase, putative
**54**	TVAG_346750	EAX97673	+1.13	2.01e-05	7.01e-03	Up	conserved hypothetical protein
**55**	TVAG_278640	EAX85446	+1.96	2.08e-05	7.14e-03	Up	conserved hypothetical protein
**56**	TVAG_369140	EAX96004	+3.89	2.14e-05	7.21e-03	Up	conserved hypothetical protein
**57**	TVAG_127770	EAX83850	+1.90	2.68e-05	8.86e-03	Up	conserved hypothetical protein
**58**	TVAG_161300	EAY12324	-5.51	2.74e-05	8.91e-03	Down	iron superoxide dismutase A, putative
**59**	TVAG_227680	EAY04651	+1.20	2.87e-05	9.19e-03	Up	conserved hypothetical protein
**60**	TVAG_165710	EAX86336	+1.33	2.97e-05	9.32e-03	Up	Inositol-tetrakisphosphate 1-kinase, putative
**61**	TVAG_008110	EAY02803	+2.87	3.07e-05	9.47e-03	Up	homeobox protein knotted-1, putative
**62**	TVAG_475160	EAY06282	+1.47	3.16e-05	9.60e-03	Up	HSP90 co-chaperone, putative
**63**	TVAG_398000	EAX82727	+2.96	3.34e-05	9.69e-03	Up	conserved hypothetical protein
**64**	TVAG_491480	EAY10291	+1.32	3.31e-05	9.69e-03	Up	conserved hypothetical protein
**65**	TVAG_453380	EAY04520	+4.55	3.31e-05	9.69e-03	Up	conserved hypothetical protein
**66**	TVAG_249310	EAY21871	+2.40	3.45e-05	9.86e-03	Up	conserved hypothetical protein
**67**	TVAG_301120	EAY12095	+2.31	3.74e-05	1.02e-02	Up	heat shock protein 70 (HSP70)-4, putative
**68**	TVAG_169010	EAY02899	+3.36	3.74e-05	1.02e-02	Up	pirin, putative
**69**	TVAG_179940	EAY00323	+4.79	3.83e-05	1.03e-02	Up	conserved hypothetical protein
**70**	TVAG_433470	EAX94939	+2.27	4.44e-05	1.18e-02	Up	aldehyde oxidase, putative
**71**	TVAG_298320	EAY04212	+1.88	4.66e-05	1.22e-02	Up	trichohyalin, putative
**72**	TVAG_034730	EAX95044	+1.60	4.81e-05	1.24e-02	Up	endoplasmin, putative
**73**	TVAG_071810	EAY23517	+3.55	4.96e-05	1.26e-02	Up	CAMK family protein kinase
**74**	TVAG_479680	EAX94203	+1.70	5.31e-05	1.33e-02	Up	2-nitropropane dioxygenase precursor, putative

**75**	TVAG_465640	EAX80269	+2.06	5.86e-05	1.45e-02	Up	tropomyosin 1 alpha chain, putative
**76**	?	EAY21878-2	+1.98	5.94e-05	1.45e-02	Up	?
**77**	TVAG_143290	EAY03029	+2.83	6.24e-05	1.51e-02	Up	Clan CA, family C19, ubiquitin hydrolase-like cysteine peptidase
**78**	TVAG_430040	EAY07640	+3.54	6.63e-05	1.58e-02	Up	conserved hypothetical protein
**79**	TVAG_185530	EAY23246	+1.55	7.04e-05	1.66e-02	Up	ankyrin repeat-containing protein, putative
**80**	TVAG_591330	EAX80117	+2.19	7.53e-05	1.71e-02	Up	synaptotagmin, putative
**81**	TVAG_276200	EAY09547	+1.51	7.48e-05	1.71e-02	Up	conserved hypothetical protein
**82**	TVAG_133370	EAY09279	+2.81	7.43e-05	1.71e-02	Up	conserved hypothetical protein
**83**	TVAG_391660	EAY20782	+1.88	8.13e-05	1.77e-02	Up	hypothetical protein
**84**	TVAG_155010	EAX90205	+1.71	8.16e-05	1.77e-02	Up	heat shock protein, putative
**85**	TVAG_265470	EAX96058	-2.50	8.06e-05	1.77e-02	Down	alcohol dehydrogenase, putative
**86**	TVAG_320780	EAX84970	+2.24	8.34e-05	1.78e-02	Up	malic enzyme, putative
**87**	TVAG_267910	EAY18758	-1.13	8.46e-05	1.78e-02	Down	calreticulin, putative
**88**	TVAG_192370	EAY20261	+1.66	8.50e-05	1.78e-02	Up	conserved hypothetical protein
**89**	TVAG_368240	EAX95423	+2.53	9.07e-05	1.87e-02	Up	axoneme-associated protein mst101, putative
**90**	TVAG_171670	EAY03069	+1.83	9.15e-05	1.87e-02	Up	heat shock protein, putative
**91**	TVAG_029100	EAY00006	+1.63	9.63e-05	1.95e-02	Up	formin domain-containing protein, putative
**92**	TVAG_144410	EAX95692	+2.30	9.74e-05	1.95e-02	Up	trichohyalin, putative
**93**	TVAG_190630	EAY19167	+1.52	1.13e-04	2.24e-02	Up	conserved hypothetical protein
**94**	TVAG_245830	EAY12351	+1.50	1.14e-04	2.24e-02	Up	conserved hypothetical protein
**95**	TVAG_368230	EAX95422	+1.30	1.19e-04	2.29e-02	Up	U3 small nucleolar ribonucleoprotein MPP10, putative

**96**	TVAG_067550	EAY16750	+1.77	1.19e-04	2.29e-02	Up	conserved hypothetical protein
**97**	TVAG_227750	EAY04658	+1.27	1.22e-04	2.31e-02	Up	Antiviral helicase SLH1, putative
**98**	TVAG_151620	EAY06355	+2.77	1.22e-04	2.31e-02	Up	heat shock protein 70 (HSP70)-4, putative
**99**	TVAG_277730	EAX96492	-2.88	1.24e-04	2.32e-02	Down	ankyrin repeat-containing protein, putative
**100**	TVAG_050340	EAY07219	+2.89	1.27e-04	2.36e-02	Up	conserved hypothetical protein
**101**	TVAG_009050	EAX74829	+3.08	1.40e-04	2.56e-02	Up	conserved hypothetical protein
**102**	TVAG_282190	EAY10619	+1.08	1.44e-04	2.57e-02	Up	conserved hypothetical protein
**103**	TVAG_304500	EAY00809	+5.36	1.42e-04	2.57e-02	Up	glycerol-3-phosphate dehydrogenase, putative
**104**	TVAG_398010	EAX82728	+1.97	1.45e-04	2.57e-02	Up	conserved hypothetical protein
**105**	TVAG_126230	EAY09462	+2.28	1.56e-04	2.74e-02	Up	conserved hypothetical protein
**106**	TVAG_101460	EAY19428	+1.37	1.59e-04	2.76e-02	Up	phosphatidylinositol 3-kinase catalytic subunit gamma, putative
**107**	TVAG_171090	EAX95220	+3.09	1.61e-04	2.76e-02	Up	malate dehydrogenase, putative
**108**	TVAG_136380	EAY19507	+1.25	1.60e-04	2.76e-02	Up	conserved hypothetical protein
**109**	TVAG_275140	EAX93399	+2.58	1.81e-04	3.02e-02	Up	conserved hypothetical protein
**110**	TVAG_189580	EAY01172	+3.23	1.80e-04	3.02e-02	Up	conserved hypothetical protein
**111**	TVAG_241160	EAX99540	+2.79	1.80e-04	3.02e-02	Up	malic enzyme, putative
**112**	TVAG_569860	EAX78379	+2.28	1.84e-04	3.05e-02	Up	synaptotagmin, putative
**113**	TVAG_433820	EAX99054	+2.48	1.90e-04	3.12e-02	Up	leucine-rich repeat protein, BspA family
**114**	TVAG_319210	EAX91375	+1.81	2.29e-04	3.64e-02	Up	conserved hypothetical protein
**115**	TVAG_091600	EAX89970	+1.62	2.30e-04	3.64e-02	Up	conserved hypothetical protein

**116**	TVAG_133920	EAX96892	+3.59	2.29e-04	3.64e-02	Up	malate dehydrogenase, putative
**117**	TVAG_216880	EAX96785	+1.17	2.28e-04	3.64e-02	Up	conserved hypothetical protein
**118**	TVAG_221660	EAY12813	+1.61	2.36e-04	3.71e-02	Up	conserved hypothetical protein
**119**	TVAG_433130	EAY19717	+2.06	2.52e-04	3.87e-02	Up	heat shock protein, putative
**120**	TVAG_291620	EAY17210	+1.67	2.49e-04	3.87e-02	Up	protein kinase inhibitor, putative
**121**	TVAG_399700	EAY11964	+2.57	2.51e-04	3.87e-02	Up	conserved hypothetical protein
**122**	TVAG_205390	EAX96252	+1.48	2.57e-04	3.91e-02	Up	GTPase mss1/trme, putative
**123**	TVAG_291640	EAY17212	+2.44	2.75e-04	4.16e-02	Up	conserved hypothetical protein
**124**	TVAG_220990	EAX91205	+1.79	2.78e-04	4.16e-02	Up	chaperone binding protein, putative
**125**	TVAG_284540	EAY05871	+1.91	2.91e-04	4.18e-02	Up	conserved hypothetical protein
**126**	TVAG_221990	EAY12845	+2.28	2.91e-04	4.18e-02	Up	conserved hypothetical protein
**127**	TVAG_481030	EAX98727	+1.74	2.85e-04	4.18e-02	Up	conserved hypothetical protein
**128**	TVAG_365540	EAY20046	-1.12	2.89e-04	4.18e-02	Down	neuroendocrine convertase 2 precursor, putative
**129**	TVAG_440600	EAX88856	+1.16	2.88e-04	4.18e-02	Up	calicylin binding protein, putative
**130**	TVAG_452240	EAY19310	+2.66	3.05e-04	4.36e-02	Up	sugar transporter, putative
**131**	TVAG_343270	EAY13498	+1.92	3.11e-04	4.40e-02	Up	conserved hypothetical protein
**132**	TVAG_328940	EAY03122	+1.85	3.23e-04	4.44e-02	Up	alcohol dehydrogenase, putative
**133**	TVAG_443320	EAY00462	-2.30	3.22e-04	4.44e-02	Down	golgin IMH1, putative
**134**	TVAG_009420	EAX89108	+2.49	3.17e-04	4.44e-02	Up	malic enzyme, putative
**135**	TVAG_387950	EAY13654	+2.68	3.26e-04	4.45e-02	Up	conserved hypothetical protein
**136**	TVAG_485580	EAX89584	+1.91	3.49e-04	4.70e-02	Up	synaptotagmin 1,2, putative
**137**	TVAG_277380	EAX91668	+1.01	3.49e-04	4.70e-02	Up	nucleotide-binding protein, putative

**138**	TVAG_108890	EAY01732	+1.66	3.64e-04	4.83e-02	Up	conserved hypothetical protein
**139**	TVAG_199270	EAY21488	+1.49	3.76e-04	4.95e-02	Up	60S ribosomal protein L7a, putative
**140**	TVAG_420470	EAY09425	+1.85	3.79e-04	4.97e-02	Up	neurofilament triplet M protein, putative

Table S3. MTZ-High vs MTZ-sensitive — significant DEGs (n = 73; 71 upregulated, 2 downregulated)

Significant differentially expressed genes (padj < 0.05 and |log2 fold change| ≥ 1) from the QC-screened DESeq2 analysis. Positive log2FC = upregulated in resistance. Ranked by padj.

Rank	TVAG_ID	Protein_ID	log2FC	p-value	padj	Direction	Product_Description
**1**	TVAG_605940	TVAG_605940	+2.92	9.26e-08	2.81e-04	Up	16S ribosomal RNA
**2**	TVAG_487750	EAY08548	+1.63	1.96e-06	1.78e-03	Up	ribosomal protein L2, putative
**3**	TVAG_364620	EAY10737	+2.06	1.98e-06	1.78e-03	Up	phosphofructokinase, putative
**4**	TVAG_005910	EAY11516	+1.79	2.35e-06	1.78e-03	Up	50S ribosomal protein L2, putative
**5**	TVAG_467840	EAY13769	+1.92	7.18e-06	4.36e-03	Up	tubulin beta chain, putative
**6**	TVAG_299380	EAY03275	+1.51	1.10e-05	5.55e-03	Up	30S ribosomal protein S11, putative
**7**	TVAG_113710	EAY18027	+1.78	1.32e-05	5.70e-03	Up	phosphoglycerate mutase, putative
**8**	TVAG_198680	EAY21429	+1.61	1.73e-05	6.46e-03	Up	40S ribosomal protein S7, putative
**9**	TVAG_206300	EAY13462	+1.56	1.92e-05	6.46e-03	Up	40S ribosomal protein S8, putative
**10**	TVAG_161100	EAY12304	+1.61	5.04e-05	1.27e-02	Up	heat shock protein 70kD, putative
**11**	TVAG_350540	EAY04379	+3.22	4.81e-05	1.27e-02	Up	alkyl hydroperoxide reductase, subunit C, putative
**12**	TVAG_498960	EAX97360	+1.50	5.01e-05	1.27e-02	Up	ribosomal protein S2, eukaryotic and archaeal form, putative
**13**	TVAG_198620	EAY21423	+1.47	6.54e-05	1.53e-02	Up	40S ribosomal protein S23, putative
**14**	TVAG_435430	EAX92491	+1.18	8.74e-05	1.88e-02	Up	40S ribosomal protein S8, putative
**15**	TVAG_199240	EAY21485	+1.37	9.29e-05	1.88e-02	Up	40S ribosomal protein S19, putative

**16**	TVAG_342830	EAY07119	+1.62	1.29e-04	2.03e-02	Up	40S ribosomal protein S14/30S ribosomal protein S11, putative
**17**	TVAG_200220	EAX88789	+1.37	1.34e-04	2.03e-02	Up	60S ribosomal protein L15, putative
**18**	TVAG_499990	TVAG_499990	+2.90	1.31e-04	2.03e-02	Up	16S ribosomal RNA
**19**	TVAG_371270	EAX90066	+1.24	1.30e-04	2.03e-02	Up	60S ribosomal protein L8, putative
**20**	TVAG_536780	TVAG_536780	+2.96	1.29e-04	2.03e-02	Up	16S ribosomal RNA
**21**	TVAG_324920	EAY01378	+1.27	1.61e-04	2.32e-02	Up	ribosomal protein S15p/S13e, putative
**22**	TVAG_159830	EAY15810	+1.43	1.86e-04	2.45e-02	Up	40S ribosomal protein S23, putative
**23**	TVAG_006250	EAY11550	+1.45	1.80e-04	2.45e-02	Up	30S ribosomal protein S8, putative
**24**	TVAG_020480	EAY02495	+1.40	2.01e-04	2.55e-02	Up	40S ribosomal protein S18, putative
**25**	TVAG_305300	EAY04781	+1.40	2.19e-04	2.56e-02	Up	40S ribosomal protein S23, putative
**26**	TVAG_199270	EAY21488	+1.19	2.13e-04	2.56e-02	Up	60S ribosomal protein L7a, putative
**27**	TVAG_066760	EAY16671	+1.32	2.37e-04	2.67e-02	Up	ribosomal protein S15p/S13e, putative
**28**	TVAG_239500	EAY20574	+1.23	2.47e-04	2.67e-02	Up	40S ribosomal protein S4, putative
**29**	TVAG_313300	EAX87627	+1.09	2.79e-04	2.92e-02	Up	40S ribosomal protein S11, putative
**30**	TVAG_145570	EAY08477	+1.44	2.89e-04	2.93e-02	Up	receptor for activated protein kinase C, putative
**31**	TVAG_128790	EAY12465	+1.60	3.10e-04	3.03e-02	Up	60S ribosomal protein L4, putative
**32**	TVAG_071920	EAY23528	+1.31	3.25e-04	3.08e-02	Up	40S ribosomal protein S23, putative
**33**	TVAG_379260	EAY22447	+1.31	3.41e-04	3.13e-02	Up	30S ribosomal protein S4e, putative
**34**	TVAG_429360	EAX98582	+1.36	3.60e-04	3.21e-02	Up	WD repeat domain, putative
**35**	TVAG_602710	TVAG_602710	+2.67	3.95e-04	3.43e-02	Up	16S ribosomal RNA
**36**	TVAG_043500	EAY03404	+1.87	4.19e-04	3.53e-02	Up	enolase, putative
**37**	TVAG_580060	TVAG_580060	+2.55	4.31e-04	3.54e-02	Up	28S ribosomal RNA
**38**	TVAG_437020	EAY20878	+1.01	4.81e-04	3.84e-02	Up	50S ribosomal protein L10e, putative
**39**	TVAG_431350	EAX85539	+1.21	5.26e-04	3.88e-02	Up	60S ribosomal protein L24, putative

**40**	TVAG_125550	EAX98411	+1.31	5.37e-04	3.88e-02	Up	ribosomal protein L3p, putative
**41**	TVAG_572270	TVAG_572270	+2.22	5.13e-04	3.88e-02	Up	16S ribosomal RNA
**42**	TVAG_199100	EAY21471	+1.17	5.01e-04	3.88e-02	Up	40S ribosomal protein S7, putative
**43**	TVAG_412690	EAY03465	-1.30	6.13e-04	3.88e-02	Down	vacuolar ATP synthase proteolipid subunit, putative
**44**	TVAG_383940	EAY01655	+1.13	6.27e-04	3.88e-02	Up	phosphoglycerate kinase, putative
**45**	TVAG_401590	EAY08305	+1.29	6.06e-04	3.88e-02	Up	60S ribosomal protein L18, putative
**46**	TVAG_108330	EAY05099	-1.92	6.07e-04	3.88e-02	Down	conserved hypothetical protein
**47**	TVAG_110520	EAY20440	+1.37	6.11e-04	3.88e-02	Up	40S ribosomal protein S27, putative
**48**	TVAG_359120	EAY11132	+1.13	6.25e-04	3.88e-02	Up	ribosomal S subunit, putative
**49**	TVAG_274830	EAY10119	+1.22	5.63e-04	3.88e-02	Up	60S ribosomal protein L7a, putative
**50**	TVAG_144030	EAX89355	+1.42	6.64e-04	4.03e-02	Up	histone H2a, putative
**51**	TVAG_075280	EAX96722	+1.46	7.03e-04	4.05e-02	Up	histone H2a, putative
**52**	TVAG_133150	EAY09257	+1.73	6.81e-04	4.05e-02	Up	ubiquitin, putative
**53**	TVAG_184530	EAY10551	+1.34	7.08e-04	4.05e-02	Up	30S ribosomal protein S13, putative
**54**	TVAG_445270	EAY12415	+1.25	7.41e-04	4.17e-02	Up	cenpa protein, putative
**55**	TVAG_151780	EAY06371	+1.17	7.64e-04	4.21e-02	Up	30S ribosomal protein S15p/S13e, putative
**56**	TVAG_070530	EAY23390	+1.15	8.61e-04	4.38e-02	Up	ribosomal protein L13, putative
**57**	TVAG_054130	EAX93808	+1.18	8.89e-04	4.38e-02	Up	60S ribosomal protein L7, putative
**58**	TVAG_131210	EAY05387	+1.32	8.94e-04	4.38e-02	Up	30S ribosomal protein S4e, putative
**59**	TVAG_486930	EAY14250	+1.02	8.72e-04	4.38e-02	Up	40S ribosomal protein S6, putative
**60**	TVAG_345360	EAY03162	+2.21	8.92e-04	4.38e-02	Up	fructose-bisphosphate aldolase, putative
**61**	TVAG_291010	EAY00395	+1.05	9.25e-04	4.46e-02	Up	60S ribosomal protein L10, putative
**62**	TVAG_401900	EAY19937	+1.02	9.63e-04	4.57e-02	Up	30S ribosomal protein S17p, putative
**63**	TVAG_542810	TVAG_542810	+2.66	1.01e-03	4.66e-02	Up	16S ribosomal RNA

**64**	TVAG_110530	EAY20441	+1.01	1.01e-03	4.66e-02	Up	40S ribosomal protein S20, putative
**65**	TVAG_147010	EAY18957	+1.95	1.06e-03	4.80e-02	Up	Derlin-2, putative
**66**	TVAG_144040	EAX89356	+1.58	1.09e-03	4.83e-02	Up	histone H2b, putative
**67**	TVAG_113720	EAY18028	+1.10	1.10e-03	4.83e-02	Up	ribosomal protein L5, putative
**68**	TVAG_456920	EAY22057	+1.33	1.20e-03	4.85e-02	Up	tubulin alpha chain, putative
**69**	TVAG_059650	EAX97900	+1.04	1.20e-03	4.85e-02	Up	ribosomal protein L3p, putative
**70**	TVAG_380930	EAX91445	+1.30	1.16e-03	4.85e-02	Up	histone H2a, putative
**71**	TVAG_417070	EAY04409	+3.23	1.19e-03	4.85e-02	Up	conserved hypothetical protein
**72**	TVAG_083260	EAY18473	+1.12	1.17e-03	4.85e-02	Up	60S ribosomal protein L17, putative
**73**	TVAG_447860	EAY16827	+1.64	1.18e-03	4.85e-02	Up	histone H2a, putative

Table S4. TDZ-High vs MTZ-sensitive — significant DEGs (n = 3; 2 upregulated, 1 downregulated)

Significant differentially expressed genes (padj < 0.05 and |log2 fold change| ≥ 1) from the QC-screened DESeq2 analysis. Positive log2FC = upregulated in resistance. Ranked by padj.

Rank	TVAG_ID	Protein_ID	log2FC	p-value	padj	Direction	Product_Description
**1**	TVAG_427000	EAX93472	+5.59	9.91e-07	1.74e-02	Up	conserved hypothetical protein
**2**	TVAG_210560	EAX91231	+3.19	1.59e-06	1.74e-02	Up	trichohyalin, putative
**3**	TVAG_517010	EAX83483	-6.87	1.52e-06	1.74e-02	Down	NAD(P)H dehydrogenase, putative

Table S5. SEC-High vs MTZ-sensitive — significant DEGs (n = 136; 134 upregulated, 2 downregulated)

Significant differentially expressed genes (padj < 0.05 and |log2 fold change| ≥ 1) from the QC-screened DESeq2 analysis. Positive log2FC = upregulated in resistance. Ranked by padj.

Rank	TVAG_ID	Protein_ID	log2FC	p-value	padj	Direction	Product_Description
**1**	TVAG_009050	EAX74829	+4.54	6.26e-09	2.29e-05	Up	conserved hypothetical protein
**2**	TVAG_576440	TVAG_576440	+3.48	6.88e-08	1.26e-04	Up	5.8S ribosomal RNA
**3**	TVAG_133150	EAY09257	+2.38	4.02e-07	4.91e-04	Up	ubiquitin, putative
**4**	TVAG_587980	TVAG_587980	+2.50	6.47e-07	5.92e-04	Up	16S ribosomal RNA

**5**	TVAG_548600	TVAG_548600	+2.15	2.92e-06	2.14e-03	Up	16S ribosomal RNA
**6**	TVAG_542850	TVAG_542850	+2.00	4.64e-06	2.83e-03	Up	16S ribosomal RNA
**7**	TVAG_096350	EAX91938	+1.76	6.28e-06	3.29e-03	Up	triosephosphate isomerase, putative
**8**	TVAG_541120	TVAG_541120	+2.04	9.89e-06	4.05e-03	Up	16S ribosomal RNA
**9**	TVAG_457460	EAY22111	+2.66	9.95e-06	4.05e-03	Up	conserved hypothetical protein
**10**	TVAG_544270	TVAG_544270	+2.02	1.20e-05	4.38e-03	Up	16S ribosomal RNA
**11**	TVAG_586540	TVAG_586540	+2.01	1.55e-05	5.12e-03	Up	16S ribosomal RNA
**12**	TVAG_540760	TVAG_540760	+1.93	1.68e-05	5.12e-03	Up	16S ribosomal RNA
**13**	TVAG_546240	TVAG_546240	+1.91	1.95e-05	5.26e-03	Up	16S ribosomal RNA
**14**	TVAG_569060	TVAG_569060	+1.96	2.11e-05	5.26e-03	Up	16S ribosomal RNA
**15**	TVAG_542290	TVAG_542290	+1.93	2.22e-05	5.26e-03	Up	16S ribosomal RNA
**16**	TVAG_110140	EAY20402	+1.45	2.30e-05	5.26e-03	Up	ubiquitin, putative
**17**	TVAG_478140	EAX89557	+1.87	2.60e-05	5.59e-03	Up	ubiquitin, putative
**18**	TVAG_528510	TVAG_528510	+1.88	3.55e-05	5.73e-03	Up	16S ribosomal RNA
**19**	TVAG_548510	TVAG_548510	+1.98	3.13e-05	5.73e-03	Up	16S ribosomal RNA
**20**	TVAG_542810	TVAG_542810	+2.39	4.37e-05	5.73e-03	Up	16S ribosomal RNA
**21**	TVAG_587640	TVAG_587640	+1.89	3.14e-05	5.73e-03	Up	16S ribosomal RNA
**22**	TVAG_589400	TVAG_589400	+1.89	3.79e-05	5.73e-03	Up	16S ribosomal RNA
**23**	TVAG_180570	EAY09082	+2.12	4.38e-05	5.73e-03	Up	conserved hypothetical protein
**24**	TVAG_539430	TVAG_539430	+1.92	3.40e-05	5.73e-03	Up	16S ribosomal RNA
**25**	TVAG_548220	TVAG_548220	+1.88	4.27e-05	5.73e-03	Up	16S ribosomal RNA
**26**	TVAG_605620	TVAG_605620	+1.88	4.15e-05	5.73e-03	Up	16S ribosomal RNA
**27**	?	EAY21878-2	+2.27	2.96e-05	5.73e-03	Up	?
**28**	TVAG_544780	TVAG_544780	+1.87	3.93e-05	5.73e-03	Up	16S ribosomal RNA
**29**	TVAG_508500	TVAG_508500	+1.83	6.46e-05	8.16e-03	Up	16S ribosomal RNA
**30**	TVAG_589790	TVAG_589790	+1.77	8.45e-05	1.03e-02	Up	16S ribosomal RNA
**31**	TVAG_571680	TVAG_571680	+1.84	8.82e-05	1.04e-02	Up	16S ribosomal RNA
**32**	TVAG_291690	EAY17217	+1.49	9.52e-05	1.09e-02	Up	60S acidic ribosomal protein P2, putative
**33**	TVAG_540960	TVAG_540960	+1.74	9.92e-05	1.10e-02	Up	16S ribosomal RNA
**34**	TVAG_544950	TVAG_544950	+1.76	1.06e-04	1.14e-02	Up	16S ribosomal RNA
**35**	TVAG_536780	TVAG_536780	+2.28	1.11e-04	1.16e-02	Up	16S ribosomal RNA
**36**	TVAG_497260	EAY08102	+1.81	1.17e-04	1.19e-02	Up	axonmeal dynein heavy chain, putative
**37**	TVAG_569780	EAX65058	+1.99	1.26e-04	1.19e-02	Up	conserved hypothetical protein
**38**	TVAG_079960	TVAG_079960	+1.77	1.21e-04	1.19e-02	Up	16S ribosomal RNA
**39**	TVAG_117900	EAY07709	+1.80	1.27e-04	1.19e-02	Up	synaptotagmin, putative

**40**	TVAG_399490	EAY11944	+2.02	1.36e-04	1.23e-02	Up	Microtubule-associated protein futsch, putative
**41**	TVAG_545530	TVAG_545530	+1.83	1.38e-04	1.23e-02	Up	16S ribosomal RNA
**42**	TVAG_578290	TVAG_578290	+1.84	1.49e-04	1.30e-02	Up	16S ribosomal RNA
**43**	TVAG_203910	EAY04087	+2.64	1.54e-04	1.30e-02	Up	myosin-2 heavy chain, non muscle, putative
**44**	TVAG_369140	EAX96004	+2.32	1.56e-04	1.30e-02	Up	conserved hypothetical protein
**45**	TVAG_577170	TVAG_577170	+1.73	1.65e-04	1.34e-02	Up	16S ribosomal RNA
**46**	TVAG_563710	TVAG_563710	+1.76	1.92e-04	1.35e-02	Up	16S ribosomal RNA
**47**	TVAG_297820	TVAG_297820	+1.70	1.93e-04	1.35e-02	Up	16S ribosomal RNA
**48**	TVAG_604520	TVAG_604520	+1.40	1.92e-04	1.35e-02	Up	28S ribosomal RNA
**49**	TVAG_420420	EAY09420	+1.42	1.86e-04	1.35e-02	Up	dj-1 protein, putative
**50**	TVAG_499990	TVAG_499990	+2.17	1.95e-04	1.35e-02	Up	16S ribosomal RNA
**51**	TVAG_585070	TVAG_585070	+1.70	1.80e-04	1.35e-02	Up	16S ribosomal RNA
**52**	TVAG_572270	TVAG_572270	+1.78	1.84e-04	1.35e-02	Up	16S ribosomal RNA
**53**	TVAG_559840	TVAG_559840	+1.72	1.93e-04	1.35e-02	Up	16S ribosomal RNA
**54**	TVAG_350540	EAY04379	+2.52	2.07e-04	1.41e-02	Up	alkyl hydroperoxide reductase, subunit C, putative
**55**	TVAG_596130	TVAG_596130	+1.70	2.11e-04	1.41e-02	Up	16S ribosomal RNA
**56**	TVAG_596370	TVAG_596370	+1.90	2.43e-04	1.59e-02	Up	16S ribosomal RNA
**57**	TVAG_387950	EAY13654	+2.51	2.71e-04	1.74e-02	Up	conserved hypothetical protein
**58**	TVAG_607470	TVAG_607470	+1.66	2.76e-04	1.74e-02	Up	16S ribosomal RNA
**59**	TVAG_006480	EAY11573	+1.71	2.83e-04	1.76e-02	Up	axonemal dynein gamma heavy chain, putative
**60**	TVAG_539080	TVAG_539080	+1.68	2.98e-04	1.78e-02	Up	16S ribosomal RNA
**61**	TVAG_479220	EAX91014	+2.02	2.98e-04	1.78e-02	Up	heat shock protein, putative
**62**	TVAG_544360	TVAG_544360	+1.64	3.00e-04	1.78e-02	Up	16S ribosomal RNA
**63**	TVAG_194870	EAX94401	-1.88	3.19e-04	1.86e-02	Down	leucine-rich repeat protein, BspA family
**64**	TVAG_501890	TVAG_501890	+1.65	3.35e-04	1.86e-02	Up	16S ribosomal RNA
**65**	TVAG_457450	EAY22110	+1.89	3.29e-04	1.86e-02	Up	KS1 protein precursor, putative
**66**	TVAG_284790	TVAG_284790	+1.64	3.34e-04	1.86e-02	Up	16S ribosomal RNA
**67**	TVAG_319090	EAY05554	+1.55	3.50e-04	1.89e-02	Up	conserved hypothetical protein
**68**	TVAG_543830	TVAG_543830	+1.69	3.47e-04	1.89e-02	Up	16S ribosomal RNA
**69**	TVAG_602710	TVAG_602710	+2.06	3.56e-04	1.89e-02	Up	16S ribosomal RNA

**70**	TVAG_184340	EAY10532	-1.18	3.81e-04	1.97e-02	Down	conserved hypothetical protein
**71**	TVAG_543620	TVAG_543620	+1.74	3.78e-04	1.97e-02	Up	16S ribosomal RNA
**72**	TVAG_583580	TVAG_583580	+1.62	3.87e-04	1.97e-02	Up	16S ribosomal RNA
**73**	TVAG_173120	EAY21054	+3.24	4.01e-04	1.99e-02	Up	abca9, putative
**74**	TVAG_539960	TVAG_539960	+1.55	4.01e-04	1.99e-02	Up	28S ribosomal RNA
**75**	TVAG_558920	TVAG_558920	+1.68	4.11e-04	2.00e-02	Up	16S ribosomal RNA
**76**	TVAG_075420	EAX96736	+1.42	4.19e-04	2.00e-02	Up	alkyl hydroperoxide reductase, subunit C, putative
**77**	TVAG_520270	TVAG_520270	+1.67	4.21e-04	2.00e-02	Up	16S ribosomal RNA
**78**	TVAG_452780	EAY19363	+1.69	4.34e-04	2.03e-02	Up	ubiquitin, putative
**79**	TVAG_159980	EAY15825	+1.42	4.38e-04	2.03e-02	Up	ubiquitin, putative
**80**	TVAG_542990	TVAG_542990	+1.64	4.89e-04	2.18e-02	Up	16S ribosomal RNA
**81**	TVAG_570690	TVAG_570690	+1.68	4.79e-04	2.18e-02	Up	16S ribosomal RNA
**82**	TVAG_557770	TVAG_557770	+1.43	4.93e-04	2.18e-02	Up	28S ribosomal RNA
**83**	TVAG_603090	TVAG_603090	+1.71	4.88e-04	2.18e-02	Up	16S ribosomal RNA
**84**	TVAG_210010	EAY15471	+1.84	5.10e-04	2.20e-02	Up	conserved hypothetical protein
**85**	TVAG_548270	TVAG_548270	+1.81	5.04e-04	2.20e-02	Up	16S ribosomal RNA
**86**	TVAG_598130	TVAG_598130	+1.75	5.45e-04	2.32e-02	Up	16S ribosomal RNA
**87**	TVAG_555490	TVAG_555490	+1.81	5.68e-04	2.39e-02	Up	16S ribosomal RNA
**88**	TVAG_536040	TVAG_536040	+2.04	5.99e-04	2.49e-02	Up	16S ribosomal RNA
**89**	TVAG_595280	TVAG_595280	+1.63	6.22e-04	2.50e-02	Up	16S ribosomal RNA
**90**	TVAG_578600	TVAG_578600	+1.61	6.20e-04	2.50e-02	Up	16S ribosomal RNA
**91**	TVAG_538390	TVAG_538390	+1.40	6.08e-04	2.50e-02	Up	28S ribosomal RNA
**92**	TVAG_552310	TVAG_552310	+1.65	6.33e-04	2.52e-02	Up	16S ribosomal RNA
**93**	TVAG_370820	EAX95578	+1.38	6.59e-04	2.57e-02	Up	60S ribosomal protein L37a, putative
**94**	TVAG_487340	EAY08510	+1.73	6.56e-04	2.57e-02	Up	galactokinase, putative
**95**	TVAG_605630	TVAG_605630	+1.60	6.96e-04	2.62e-02	Up	16S ribosomal RNA
**96**	TVAG_110520	EAY20440	+1.22	6.85e-04	2.62e-02	Up	40S ribosomal protein S27, putative
**97**	TVAG_589050	TVAG_589050	+1.79	7.08e-04	2.62e-02	Up	28S ribosomal RNA
**98**	TVAG_383100	TVAG_383100	+1.55	6.90e-04	2.62e-02	Up	16S ribosomal RNA
**99**	TVAG_538910	TVAG_538910	+1.81	7.06e-04	2.62e-02	Up	16S ribosomal RNA
**100**	TVAG_462090	TVAG_462090	+1.61	7.69e-04	2.79e-02	Up	16S ribosomal RNA
**101**	TVAG_601650	TVAG_601650	+1.70	7.68e-04	2.79e-02	Up	16S ribosomal RNA
**102**	TVAG_564960	TVAG_564960	+1.70	7.94e-04	2.83e-02	Up	16S ribosomal RNA
**103**	TVAG_497370	EAY08113	+1.37	7.95e-04	2.83e-02	Up	triosephosphate isomerase, putative
**104**	TVAG_539720	TVAG_539720	+1.54	8.50e-04	3.00e-02	Up	16S ribosomal RNA

**105**	TVAG_582510	TVAG_582510	+1.58	8.79e-04	3.07e-02	Up	16S ribosomal RNA
**106**	TVAG_520860	TVAG_520860	+1.56	9.15e-04	3.15e-02	Up	28S ribosomal RNA
**107**	TVAG_552620	TVAG_552620	+1.67	9.48e-04	3.19e-02	Up	16S ribosomal RNA
**108**	TVAG_068600	TVAG_068600	+1.69	9.91e-04	3.30e-02	Up	16S ribosomal RNA
**109**	TVAG_337230	EAX92303	+1.63	1.01e-03	3.32e-02	Up	superoxide dismutase [fe], putative
**110**	TVAG_452020	EAY19288	+1.27	1.06e-03	3.43e-02	Up	conserved hypothetical protein
**111**	TVAG_151620	EAY06355	+1.92	1.05e-03	3.43e-02	Up	heat shock protein 70 (HSP70)-4, putative
**112**	TVAG_298320	EAY04212	+1.89	1.07e-03	3.45e-02	Up	trichohyalin, putative
**113**	TVAG_212940	TVAG_212940	+1.50	1.13e-03	3.56e-02	Up	16S ribosomal RNA
**114**	TVAG_542820	TVAG_542820	+1.57	1.13e-03	3.56e-02	Up	28S ribosomal RNA
**115**	TVAG_440130	EAX96997	+1.41	1.17e-03	3.66e-02	Up	axonemal dynein beta heavy chain, putative
**116**	TVAG_021170	EAY20172	+1.37	1.18e-03	3.66e-02	Up	conserved hypothetical protein
**117**	TVAG_475220	EAY06288	+1.42	1.22e-03	3.77e-02	Up	glyceraldehyde 3-phosphate dehydrogenase, putative
**118**	TVAG_556860	TVAG_556860	+1.53	1.31e-03	3.98e-02	Up	16S ribosomal RNA
**119**	TVAG_008840	EAX95873	+1.67	1.39e-03	4.17e-02	Up	conserved hypothetical protein
**120**	TVAG_544610	TVAG_544610	+1.50	1.48e-03	4.41e-02	Up	16S ribosomal RNA
**121**	TVAG_328180	EAX90717	+1.54	1.52e-03	4.43e-02	Up	proteasome-activating nucleotidase, putative
**122**	TVAG_147790	EAX91132	+1.23	1.53e-03	4.43e-02	Up	cysteine/methionine metabolism pyridoxal-5-phosphate enzymes, putative
**123**	TVAG_095450	TVAG_095450	+1.28	1.53e-03	4.43e-02	Up	28S ribosomal RNA
**124**	TVAG_396380	TVAG_396380	+1.62	1.56e-03	4.47e-02	Up	16S ribosomal RNA
**125**	TVAG_585860	TVAG_585860	+1.52	1.64e-03	4.66e-02	Up	16S ribosomal RNA
**126**	TVAG_110540	EAY20442	+1.44	1.68e-03	4.74e-02	Up	ubiquitin, putative
**127**	TVAG_607440	TVAG_607440	+1.41	1.75e-03	4.85e-02	Up	28S ribosomal RNA
**128**	TVAG_207430	EAY00840	+1.09	1.78e-03	4.85e-02	Up	60S ribosomal protein L36e, putative

**129**	TVAG_029100	EAY00006	+1.44	1.76e-03	4.85e-02	Up	formin domain-containing protein, putative
**130**	TVAG_587560	TVAG_587560	+1.45	1.78e-03	4.85e-02	Up	28S ribosomal RNA
**131**	TVAG_550360	TVAG_550360	+1.50	1.83e-03	4.86e-02	Up	28S ribosomal RNA
**132**	TVAG_176780	TVAG_176780	+1.50	1.84e-03	4.86e-02	Up	16S ribosomal RNA
**133**	TVAG_528610	TVAG_528610	+1.26	1.84e-03	4.86e-02	Up	28S ribosomal RNA
**134**	TVAG_591330	EAX80117	+1.86	1.88e-03	4.88e-02	Up	synaptotagmin, putative
**135**	TVAG_538210	TVAG_538210	+1.66	1.87e-03	4.88e-02	Up	16S ribosomal RNA
**136**	TVAG_604820	TVAG_604820	+2.09	1.93e-03	4.93e-02	Up	16S ribosomal RNA

Table S6. WGCNA module–trait correlations

*Pearson correlation coefficient (with p-value and significance code) between each WGCNA module eigengene and each resistance trait, from the rRNA-filtered v2 WGCNA analysis. Continuous traits: MTZ/TDZ/SEC MLC. Categorical traits: High-/Inter-/Low-resistance and MTZ-sensitive status. Significance code: *** p<0.001, ** p<0.01, * p<0.05. Modules are ordered by ascending MTZ_MLC p-value.*

Module	MTZ MLC	TDZ MLC	SEC MLC	High-resis	Inter-resis	Low-resis	MTZ-sens
**magenta**	+0.46 (0.013)*	+0.31 (0.104)	+0.31 (0.101)	+0.46 (0.012)*	+0.12 (0.521)	-0.23 (0.222)	-0.22 (0.247)
**darkturquoise**	-0.39 (0.034)*	-0.17 (0.389)	-0.41 (0.029)*	-0.34 (0.067)	-0.11 (0.576)	+0.03 (0.884)	+0.30 (0.118)
**darkgreen**	+0.33 (0.082)	+0.27 (0.162)	+0.37 (0.046)*	+0.29 (0.126)	+0.08 (0.695)	+0.12 (0.540)	-0.35 (0.059)
**brown**	-0.32 (0.085)	+0.09 (0.626)	-0.19 (0.317)	-0.39 (0.036)*	+0.07 (0.713)	+0.19 (0.322)	+0.06 (0.755)
**cyan**	+0.32 (0.087)	+0.38 (0.041)*	+0.38 (0.045)*	+0.14 (0.465)	+0.24 (0.218)	+0.24 (0.205)	-0.47 (0.010)**
**darkred**	-0.31 (0.102)	-0.10 (0.615)	-0.23 (0.236)	-0.28 (0.142)	-0.12 (0.529)	+0.01 (0.951)	+0.28 (0.149)
**salmon**	-0.27 (0.154)	-0.15 (0.427)	-0.13 (0.499)	-0.28 (0.145)	-0.05 (0.796)	+0.16 (0.418)	+0.10 (0.596)
**lightyellow**	+0.27 (0.156)	+0.24 (0.205)	+0.30 (0.111)	+0.04 (0.825)	+0.67 (0.000)***	-0.30 (0.113)	-0.29 (0.125)
**turquoise**	-0.25 (0.187)	-0.04 (0.839)	-0.28 (0.144)	-0.28 (0.141)	+0.19 (0.314)	-0.03 (0.867)	+0.07 (0.704)
**red**	-0.22 (0.249)	+0.20 (0.310)	-0.12 (0.552)	-0.28 (0.143)	+0.07 (0.701)	+0.08 (0.680)	+0.07 (0.714)
**midnightblue**	-0.21 (0.270)	+0.14 (0.474)	-0.13 (0.495)	-0.28 (0.144)	+0.11 (0.565)	+0.18 (0.361)	-0.03 (0.858)
**lightgreen**	-0.20 (0.291)	-0.24 (0.201)	-0.13 (0.498)	-0.14 (0.478)	-0.15 (0.435)	+0.06 (0.776)	+0.16 (0.396)
**green**	-0.18 (0.352)	-0.10 (0.592)	-0.06 (0.773)	-0.13 (0.498)	-0.11 (0.557)	+0.22 (0.253)	-0.00 (0.994)
**tan**	+0.17 (0.383)	+0.13 (0.513)	+0.14 (0.479)	+0.13 (0.496)	-0.04 (0.828)	+0.24 (0.208)	-0.25 (0.183)
**lightcyan**	-0.11 (0.555)	-0.02 (0.915)	-0.06 (0.749)	-0.14 (0.455)	+0.23 (0.235)	-0.14 (0.482)	+0.04 (0.845)
**grey60**	+0.11 (0.562)	-0.04 (0.855)	+0.19 (0.318)	-0.10 (0.616)	+0.35 (0.061)	+0.29 (0.131)	-0.43 (0.020)*
**black**	+0.11 (0.575)	-0.20 (0.295)	+0.15 (0.450)	+0.01 (0.972)	+0.36 (0.053)	-0.10 (0.590)	-0.20 (0.311)
**purple**	+0.10 (0.613)	-0.04 (0.835)	-0.06 (0.774)	+0.11 (0.556)	+0.09 (0.651)	-0.11 (0.566)	-0.05 (0.777)
**royalblue**	-0.09 (0.629)	-0.35 (0.065)	-0.20 (0.301)	-0.04 (0.855)	-0.02 (0.908)	-0.09 (0.641)	+0.11 (0.553)
**greenyellow**	+0.09 (0.638)	-0.10 (0.590)	-0.07 (0.706)	+0.12 (0.531)	+0.10 (0.616)	-0.27 (0.150)	+0.07 (0.737)
**pink**	+0.07 (0.720)	+0.18 (0.338)	+0.21 (0.266)	+0.02 (0.935)	+0.02 (0.900)	+0.05 (0.808)	-0.07 (0.728)
**blue**	-0.07 (0.729)	+0.12 (0.521)	+0.05 (0.795)	-0.11 (0.579)	-0.05 (0.787)	+0.21 (0.278)	-0.05 (0.777)
**grey**	+0.06 (0.773)	+0.05 (0.812)	+0.07 (0.718)	+0.06 (0.760)	+0.03 (0.865)	+0.07 (0.724)	-0.12 (0.531)

**darkgrey**	+0.02 (0.916)	-0.18 (0.350)	+0.12 (0.522)	-0.04 (0.832)	+0.19 (0.313)	-0.16 (0.409)	+0.01 (0.956)
**yellow**	-0.01 (0.976)	+0.13 (0.498)	+0.02 (0.922)	-0.09 (0.645)	+0.39 (0.036)*	-0.06 (0.773)	-0.19 (0.326)

Table S7. WGCNA hub genes for the four MLC-trait-significant modules

Hub genes (module membership kME ≥ 0.8) for each of the four WGCNA modules significantly correlated with a continuous MLC trait. GS = gene significance (correlation of the individual gene with the trait). Sorted by kME within each module.

Module magenta (265 genes; 50 hubs at kME ≥ 0.8)

Rank	TVAG_ID	Protein_ID	kME	GS_MTZ_MLC	GS_TDZ_MLC	GS_SEC_MLC	Product_Description
**1**	TVAG_054130	EAX93808	0.974	+0.413	+0.269	+0.260	60S ribosomal protein L7, putative
**2**	TVAG_462690	EAY18583	0.966	+0.405	+0.232	+0.351	30S ribosomal protein S3, putative
**3**	TVAG_498960	EAX97360	0.962	+0.503	+0.367	+0.388	ribosomal protein S2, eukaryotic and archaeal form, putative
**4**	TVAG_246200	EAY12386	0.960	+0.385	+0.298	+0.282	ribosomal protein S7, putative
**5**	TVAG_490240	EAY01436	0.960	+0.415	+0.283	+0.230	ribosomal protein L5, putative
**6**	TVAG_145570	EAY08477	0.958	+0.460	+0.308	+0.356	receptor for activated protein kinase C, putative
**7**	TVAG_013060	EAY21541	0.954	+0.406	+0.216	+0.235	60S ribosomal protein L3, putative
**8**	TVAG_074610	EAY12633	0.954	+0.350	+0.113	+0.328	60S ribosomal protein L10, putative
**9**	TVAG_437020	EAY20878	0.953	+0.450	+0.304	+0.298	50S ribosomal protein L10e, putative
**10**	TVAG_395470	EAY01300	0.949	+0.388	+0.287	+0.256	60S ribosomal protein L18, putative
**11**	TVAG_206300	EAY13462	0.949	+0.531	+0.369	+0.332	40S ribosomal protein S8, putative
**12**	TVAG_151920	EAY06385	0.948	+0.478	+0.276	+0.253	WD repeat domain, putative
**13**	TVAG_464120	EAY13278	0.946	+0.404	+0.255	+0.291	30S ribosomal protein S11, putative
**14**	TVAG_121100	EAY23772	0.946	+0.428	+0.286	+0.312	60S ribosomal protein L18, putative
**15**	TVAG_274830	EAY10119	0.945	+0.396	+0.266	+0.332	60S ribosomal protein L7a, putative

**16**	TVAG_484060	EAY10495	0.945	+0.367	+0.240	+0.200	ribosomal protein L13, putative
**17**	TVAG_113720	EAY18028	0.944	+0.468	+0.253	+0.354	ribosomal protein L5, putative
**18**	TVAG_239500	EAY20574	0.939	+0.504	+0.395	+0.316	40S ribosomal protein S4, putative
**19**	TVAG_454000	EAY17572	0.939	+0.365	+0.139	+0.382	ribosomal protein L5, putative
**20**	TVAG_459150	EAY11843	0.938	+0.406	+0.379	+0.271	ribosomal protein S15p/S13e, putative
**21**	TVAG_306310	EAY18109	0.936	+0.412	+0.267	+0.256	60S ribosomal protein L27e, putative
**22**	TVAG_314710	EAY03941	0.934	+0.363	+0.278	+0.152	30S ribosomal protein S3, putative
**23**	TVAG_291010	EAY00395	0.932	+0.478	+0.333	+0.242	60S ribosomal protein L10, putative
**24**	TVAG_359120	EAY11132	0.932	+0.429	+0.309	+0.309	ribosomal S subunit, putative
**25**	TVAG_429360	EAX98582	0.931	+0.461	+0.305	+0.299	WD repeat domain, putative
**26**	TVAG_106800	EAY11785	0.931	+0.351	+0.196	+0.370	30S ribosomal protein S3, putative
**27**	TVAG_184530	EAY10551	0.929	+0.427	+0.384	+0.187	30S ribosomal protein S13, putative
**28**	TVAG_110890	EAY20476	0.929	+0.350	+0.214	+0.163	60S ribosomal protein L13a, putative
**29**	TVAG_164550	EAY13345	0.928	+0.346	+0.196	+0.332	60S ribosomal protein L4, putative
**30**	TVAG_194850	EAX94399	0.927	+0.398	+0.352	+0.116	60S ribosomal protein L11, putative
**31**	TVAG_401590	EAY08305	0.926	+0.474	+0.334	+0.254	60S ribosomal protein L18, putative
**32**	TVAG_414510	EAX96970	0.926	+0.389	+0.165	+0.301	40S ribosomal protein S3a, putative
**33**	TVAG_299380	EAY03275	0.923	+0.491	+0.378	+0.349	30S ribosomal protein S11, putative
**34**	TVAG_324920	EAY01378	0.923	+0.409	+0.342	+0.311	ribosomal protein S15p/S13e, putative
**35**	TVAG_337950	EAX96363	0.921	+0.410	+0.271	+0.204	60S ribosomal protein L27e, putative
**36**	TVAG_006170	EAY11542	0.920	+0.397	+0.234	+0.215	60S ribosomal protein L19, putative
**37**	TVAG_071920	EAY23528	0.919	+0.481	+0.403	+0.261	40S ribosomal protein S23, putative

**38**	TVAG_474410	EAY04235	0.919	+0.379	+0.142	+0.379	40S ribosomal protein sa, putative
**39**	TVAG_189110	EAY00695	0.915	+0.363	+0.147	+0.250	60S ribosomal protein L4, putative
**40**	TVAG_152720	EAX90705	0.914	+0.338	+0.266	+0.150	40S ribosomal protein S14, putative
**41**	TVAG_125550	EAX98411	0.913	+0.431	+0.229	+0.331	ribosomal protein L3p, putative
**42**	TVAG_297470	EAY17068	0.910	+0.335	+0.267	+0.218	60S ribosomal protein L9, putative
**43**	TVAG_198960	EAY21457	0.910	+0.279	+0.185	+0.112	60S ribosomal protein L9, putative
**44**	TVAG_164160	EAY13307	0.909	+0.380	+0.258	+0.270	40S ribosomal protein S8, putative
**45**	TVAG_198680	EAY21429	0.909	+0.513	+0.395	+0.398	40S ribosomal protein S7, putative
**46**	TVAG_438370	EAY02246	0.908	+0.303	+0.191	+0.163	60S ribosomal protein L13, putative
**47**	TVAG_342830	EAY07119	0.907	+0.493	+0.412	+0.341	40S ribosomal protein S14/30S ribosomal protein S11, putative
**48**	TVAG_348090	EAY16499	0.907	+0.368	+0.210	+0.144	40S ribosomal protein S3a, putative
**49**	TVAG_371270	EAX90066	0.905	+0.460	+0.365	+0.136	60S ribosomal protein L8, putative
**50**	TVAG_121550	EAY10751	0.905	+0.398	+0.215	+0.380	60S ribosomal protein L27a, putative

Module darkturquoise (40 genes; 40 hubs at kME ≥ 0.8)

Rank	TVAG_ID	Protein_ID	kME	GS_MTZ_MLC	GS_TDZ_MLC	GS_SEC_MLC	Product_Description
**1**	TVAG_339770	EAY01349	0.933	-0.414	-0.167	-0.357	conserved hypothetical protein
**2**	TVAG_110080	EAY20396	0.912	-0.418	-0.088	-0.465	conserved hypothetical protein
**3**	TVAG_120570	EAY23719	0.892	-0.489	-0.181	-0.631	conserved hypothetical protein
**4**	TVAG_295100	EAY18858	0.880	-0.466	-0.251	-0.305	conserved hypothetical protein
**5**	TVAG_011190	EAY17893	0.875	-0.236	-0.077	-0.299	conserved hypothetical protein
**6**	TVAG_051580	EAY02026	0.846	-0.268	+0.036	-0.328	conserved hypothetical protein

**7**	TVAG_007250	EAY00195	0.844	-0.208	+0.069	-0.234	conserved hypothetical protein
**8**	TVAG_375200	EAX95811	0.841	-0.254	-0.007	-0.263	conserved hypothetical protein
**9**	TVAG_000870	EAY07803	0.823	-0.305	-0.272	-0.396	conserved hypothetical protein
**10**	TVAG_275800	EAY02306	0.808	-0.391	-0.186	-0.286	conserved hypothetical protein
**11**	TVAG_401440	EAY08290	0.803	-0.181	+0.161	-0.162	conserved hypothetical protein
**12**	TVAG_139340	EAY12554	0.797	-0.303	-0.079	-0.335	CMGC family protein kinase
**13**	TVAG_390490	EAY04279	0.795	-0.309	-0.082	-0.381	AP-1 complex subunit beta-1, putative
**14**	TVAG_379050	EAY22426	0.793	-0.255	-0.220	-0.181	conserved hypothetical protein
**15**	TVAG_074490	EAY12621	0.792	-0.380	-0.302	-0.309	Clan AD, family A22, presenilin-like aspartic peptidase
**16**	TVAG_057840	EAX94283	0.784	-0.143	-0.007	-0.364	conserved hypothetical protein
**17**	TVAG_459200	EAY11848	0.782	-0.418	-0.166	-0.537	conserved hypothetical protein
**18**	TVAG_033610	EAX87969	0.780	-0.152	-0.158	-0.330	conserved hypothetical protein
**19**	TVAG_181940	EAX99337	0.765	-0.232	-0.229	-0.163	conserved hypothetical protein
**20**	TVAG_365110	EAX87838	0.742	-0.247	-0.026	-0.312	ribokinase, putative
**21**	TVAG_441490	EAX94330	0.740	-0.228	-0.149	-0.512	conserved hypothetical protein
**22**	TVAG_170020	EAY17690	0.735	-0.373	+0.003	-0.329	1-acyl-sn-glycerol-3-phosphate acyltransferase eta, putative
**23**	TVAG_327520	EAX92443	0.712	-0.362	-0.288	-0.118	conserved hypothetical protein
**24**	TVAG_607170	EAX68037	0.707	-0.457	-0.209	-0.438	conserved hypothetical protein
**25**	TVAG_035520	EAY22529	0.707	-0.367	-0.116	-0.246	Clan CD, family C13, legumain-like cysteine peptidase
**26**	TVAG_493020	EAX91334	0.707	-0.302	-0.064	-0.313	conserved hypothetical protein
**27**	TVAG_280020	EAX97701	0.704	-0.438	-0.280	-0.253	conserved hypothetical protein

**28**	TVAG_277700	EAX96489	0.704	-0.290	-0.196	-0.089	conserved hypothetical protein
**29**	TVAG_473340	EAY03710	0.701	-0.121	-0.163	-0.256	STE family protein kinase
**30**	TVAG_379480	EAY11366	0.694	-0.267	-0.157	-0.354	clathrin coat assembly protein ap-1, putative
**31**	TVAG_482290	EAY09955	0.690	-0.138	-0.238	-0.221	small GTPase rabh, putative
**32**	TVAG_306410	EAY18119	0.683	-0.309	+0.050	-0.239	conserved hypothetical protein
**33**	TVAG_371590	EAY13686	0.657	-0.291	-0.371	-0.177	Submandibular gland secretory Glx-rich protein CA precursor, putative
**34**	TVAG_362370	EAY11857	0.631	-0.255	+0.050	-0.476	conserved hypothetical protein
**35**	TVAG_317710	EAY00486	0.623	-0.333	-0.142	-0.140	conserved hypothetical protein
**36**	TVAG_113940	EAY18050	0.618	-0.034	+0.011	-0.091	conserved hypothetical protein
**37**	TVAG_157790	EAX97794	0.595	-0.294	-0.316	-0.349	3-oxo-5-alpha-steroid 4-dehydrogenase, putative
**38**	TVAG_040010	EAY04949	0.581	-0.463	-0.155	-0.407	zinc-iron transporter, putative
**39**	TVAG_167270	EAY21352	0.553	-0.277	-0.135	-0.251	leucine-rich repeat protein, BspA family
**40**	TVAG_579070	EAX85090	0.502	-0.110	+0.061	-0.139	leucine-rich repeat protein, BspA family

Module lightyellow (98 genes; 50 hubs at kME ≥ 0.8)

Rank	TVAG_ID	Protein_ID	kME	GS_MTZ_MLC	GS_TDZ_MLC	GS_SEC_MLC	Product_Description
**1**	TVAG_132230	EAX93506	0.953	+0.269	+0.317	+0.253	conserved hypothetical protein
**2**	TVAG_227950	EAX81953	0.944	+0.173	+0.237	+0.265	conserved hypothetical protein
**3**	TVAG_415490	EAX88457	0.941	+0.218	+0.152	+0.296	conserved hypothetical protein
**4**	TVAG_097870	EAY13217	0.928	+0.202	+0.176	+0.232	conserved hypothetical protein
**5**	TVAG_188320	EAY15781	0.919	+0.284	+0.310	+0.267	conserved hypothetical protein

**6**	TVAG_227940	EAX81952	0.911	+0.187	+0.117	+0.314	conserved hypothetical protein
**7**	TVAG_259370	EAY09856	0.903	+0.212	+0.220	+0.204	conserved hypothetical protein
**8**	TVAG_189610	EAY01175	0.897	+0.380	+0.395	+0.378	conserved hypothetical protein
**9**	TVAG_233640	EAX94113	0.892	+0.318	+0.365	+0.276	conserved hypothetical protein
**10**	TVAG_345530	EAX90993	0.889	+0.325	+0.151	+0.317	conserved hypothetical protein
**11**	TVAG_306580	EAY18136	0.886	+0.272	+0.289	+0.189	conserved hypothetical protein
**12**	TVAG_554600	EAX68806	0.878	+0.288	+0.334	+0.317	conserved hypothetical protein
**13**	TVAG_399800	EAX75545	0.869	+0.387	+0.314	+0.307	conserved hypothetical protein
**14**	TVAG_380280	EAY14918	0.864	+0.346	+0.200	+0.390	conserved hypothetical protein
**15**	TVAG_399260	EAY11921	0.863	+0.203	+0.180	+0.250	conserved hypothetical protein
**16**	TVAG_380670	EAX90771	0.856	+0.236	+0.243	+0.226	conserved hypothetical protein
**17**	TVAG_189600	EAY01174	0.847	+0.306	+0.338	+0.368	conserved hypothetical protein
**18**	TVAG_380290	EAY14919	0.845	+0.433	+0.155	+0.497	conserved hypothetical protein
**19**	TVAG_496810	EAX93359	0.838	+0.108	+0.233	+0.209	conserved hypothetical protein
**20**	TVAG_243900	EAX78477	0.837	+0.352	+0.366	+0.291	conserved hypothetical protein
**21**	TVAG_547940	EAX79580	0.836	+0.277	+0.246	+0.210	conserved hypothetical protein
**22**	TVAG_189580	EAY01172	0.833	+0.389	+0.432	+0.405	conserved hypothetical protein
**23**	TVAG_391660	EAY20782	0.830	+0.422	+0.271	+0.295	hypothetical protein
**24**	TVAG_169190	EAX77358	0.824	+0.238	+0.304	+0.144	conserved hypothetical protein
**25**	TVAG_503660	EAX78014	0.822	+0.264	+0.173	+0.271	conserved hypothetical protein
**26**	TVAG_398000	EAX82727	0.822	+0.117	+0.093	+0.372	conserved hypothetical protein
**27**	TVAG_042320	EAY03580	0.821	+0.025	-0.006	+0.102	conserved hypothetical protein
**28**	TVAG_284540	EAY05871	0.816	+0.274	+0.303	+0.253	conserved hypothetical protein
**29**	TVAG_398010	EAX82728	0.812	+0.129	+0.176	+0.204	conserved hypothetical protein

**30**	TVAG_158700	EAY11643	0.811	+0.061	+0.123	+0.222	conserved hypothetical protein
**31**	TVAG_110900	EAY20477	0.806	+0.423	+0.179	+0.490	conserved hypothetical protein
**32**	TVAG_317190	EAX92661	0.806	+0.052	+0.031	+0.118	conserved hypothetical protein
**33**	TVAG_402770	EAY20021	0.805	+0.164	+0.166	+0.266	conserved hypothetical protein
**34**	TVAG_166550	EAY21280	0.800	+0.085	+0.087	+0.015	conserved hypothetical protein
**35**	TVAG_291650	EAY17213	0.796	+0.100	+0.075	+0.344	conserved hypothetical protein
**36**	TVAG_372760	EAY02130	0.786	+0.156	+0.185	+0.141	conserved hypothetical protein
**37**	TVAG_500630	EAX75018	0.782	+0.243	+0.173	+0.317	conserved hypothetical protein
**38**	TVAG_546550	EAX74772	0.777	+0.283	+0.214	+0.207	conserved hypothetical protein
**39**	TVAG_275140	EAX93399	0.769	+0.024	-0.134	+0.158	conserved hypothetical protein
**40**	TVAG_278640	EAX85446	0.765	+0.165	+0.122	+0.050	conserved hypothetical protein
**41**	TVAG_232340	EAX81728	0.764	+0.166	+0.128	+0.243	conserved hypothetical protein
**42**	TVAG_453380	EAY04520	0.762	+0.337	+0.286	+0.184	conserved hypothetical protein
**43**	TVAG_320780	EAX84970	0.757	+0.435	+0.359	+0.288	malic enzyme, putative
**44**	TVAG_291640	EAY17212	0.754	-0.010	-0.063	+0.225	conserved hypothetical protein
**45**	TVAG_104020	EAX93683	0.754	+0.318	+0.284	+0.385	conserved hypothetical protein
**46**	TVAG_151660	EAY06359	0.745	+0.388	+0.183	+0.572	conserved hypothetical protein
**47**	TVAG_098470	EAY09712	0.740	+0.300	-0.009	+0.418	conserved hypothetical protein
**48**	TVAG_166540	EAY21279	0.733	+0.096	+0.229	+0.116	conserved hypothetical protein
**49**	TVAG_127770	EAX83850	0.729	+0.492	+0.318	+0.376	conserved hypothetical protein
**50**	TVAG_250660	EAY04306	0.728	+0.085	+0.076	+0.091	conserved hypothetical protein

Module cyan (211 genes; 50 hubs at kME ≥ 0.8)

Rank	TVAG_ID	Protein_ID	kME	GS_MTZ_MLC	GS_TDZ_MLC	GS_SEC_MLC	Product_Description
1	TVAG_063660	EAY03853	0.906	+0.123	+0.232	+0.235	conserved hypothetical protein
2	TVAG_351730	EAY14125	0.898	+0.321	+0.416	+0.274	conserved hypothetical protein
3	TVAG_341360	EAY16242	0.890	+0.355	+0.426	+0.383	centromeric histone H3 htr12, putative
4	TVAG_329270	EAY10040	0.887	+0.283	+0.413	+0.293	histone H2b, putative
5	TVAG_286530	EAY05475	0.882	+0.415	+0.389	+0.395	ankyrin repeat-containing protein, putative
6	TVAG_110640	EAY20452	0.870	+0.142	+0.169	+0.235	conserved hypothetical protein
7	TVAG_508140	EAX84555	0.869	+0.207	+0.316	+0.299	conserved hypothetical protein
8	TVAG_407720	EAX93588	0.861	+0.255	+0.325	+0.347	conserved hypothetical protein
9	TVAG_026380	EAY14317	0.860	+0.317	+0.382	+0.394	histone H2a, putative
10	TVAG_061300	EAX90022	0.858	+0.059	+0.125	+0.307	conserved hypothetical protein
11	TVAG_026390	EAY14318	0.854	+0.271	+0.412	+0.332	histone H2b, putative
12	TVAG_147010	EAY18957	0.853	+0.439	+0.420	+0.258	Derlin-2, putative
13	TVAG_230180	EAY01414	0.852	+0.302	+0.332	+0.293	UDP-glucose 6-dehydrogenase, putative
14	TVAG_144040	EAX89356	0.850	+0.476	+0.512	+0.346	histone H2b, putative
15	TVAG_196890	EAX97060	0.849	+0.175	+0.178	+0.335	r2r3-MYB transcription factor, putative
16	TVAG_365790	EAY20071	0.836	+0.238	+0.282	+0.438	conserved hypothetical protein
17	TVAG_264700	EAX98421	0.833	+0.160	+0.147	+0.190	ubiquitin, putative
18	TVAG_466130	EAY02753	0.833	+0.130	+0.236	+0.323	conserved hypothetical protein
19	TVAG_038430	EAY14736	0.829	+0.034	+0.207	+0.193	conserved hypothetical protein
20	TVAG_414560	EAX84465	0.820	+0.272	+0.346	+0.149	DTDP-glucose 4,6-dehydratase, putative
21	TVAG_124850	EAY07512	0.819	+0.220	+0.201	+0.192	conserved hypothetical protein
22	TVAG_329280	EAY10041	0.818	+0.344	+0.361	+0.390	histone H2a, putative
23	TVAG_137920	EAY09800	0.816	+0.290	+0.362	+0.285	profilin
24	TVAG_417010	EAY04403	0.812	+0.279	+0.413	+0.423	conserved hypothetical protein

**25**	TVAG_417070	EAY04409	0.810	+0.250	+0.360	+0.433	conserved hypothetical protein
**26**	TVAG_318200	EAX88095	0.808	+0.226	+0.356	+0.236	r2r3-MYB transcription factor, putative
**27**	TVAG_453190	EAY04502	0.806	+0.444	+0.481	+0.238	STE family protein kinase
**28**	TVAG_447860	EAY16827	0.803	+0.333	+0.445	+0.333	histone H2a, putative
**29**	TVAG_414250	EAY09773	0.796	+0.162	+0.210	+0.242	26S proteasome non-ATPase regulatory subunit, putative
**30**	TVAG_034170	EAY06238	0.796	+0.181	+0.236	+0.256	conserved hypothetical protein
**31**	TVAG_239990	EAY08639	0.792	+0.331	+0.424	+0.204	malate dehydrogenase, putative
**32**	TVAG_341350	EAY16241	0.792	+0.306	+0.383	+0.296	histone H4, putative
**33**	TVAG_101620	EAY19444	0.791	+0.265	+0.164	+0.219	NAD dependent epimerase/dehydratase, putative
**34**	TVAG_041960	EAY03544	0.789	+0.182	+0.233	+0.222	conserved hypothetical protein
**35**	TVAG_195300	EAX94069	0.788	+0.354	+0.356	+0.295	histone H2b, putative
**36**	TVAG_193820	EAY03311	0.787	+0.465	+0.306	+0.542	Circumsporozoite protein precursor, putative
**37**	TVAG_073880	EAY09022	0.787	+0.387	+0.525	+0.329	CAMK family protein kinase
**38**	TVAG_548750	EAX82272	0.786	+0.252	+0.301	+0.197	conserved hypothetical protein
**39**	TVAG_480220	EAX94741	0.784	+0.125	+0.207	+0.200	conserved hypothetical protein
**40**	TVAG_416980	EAY04400	0.780	+0.323	+0.269	+0.591	conserved hypothetical protein
**41**	TVAG_452730	EAY19358	0.779	+0.390	+0.399	+0.458	cenpa protein, putative
**42**	TVAG_000540	EAY07770	0.779	+0.392	+0.402	+0.447	r2r3-MYB transcription factor, putative
**43**	TVAG_286160	EAX93777	0.779	+0.075	+0.039	+0.102	conserved hypothetical protein
**44**	TVAG_440420	EAY01216	0.778	+0.262	+0.460	+0.264	ubiquitin-protein ligase, putative
**45**	TVAG_402220	EAY19968	0.778	+0.128	+0.112	+0.216	conserved hypothetical protein
**46**	TVAG_475080	EAY06274	0.777	+0.376	+0.389	+0.453	conserved hypothetical protein
**47**	TVAG_212040	EAY07565	0.777	+0.351	+0.287	+0.252	sua5 protein, putative
**48**	TVAG_211260	EAY06676	0.773	+0.315	+0.427	+0.307	histone H3, putative
**49**	?	EAY03311-2	0.770	+0.463	+0.244	+0.564	?

**50**	TVAG_452180	EAY19304	0.769	+0.180	+0.259	+0.243	1-O-acylceramide synthase, putative
